# Subtype-specific structural features of the hearing loss–associated human P2X2 receptor

**DOI:** 10.1073/pnas.2417753122

**Published:** 2025-09-12

**Authors:** Franka G. Westermann, Adam C. Oken, Philip K. E. Granith, Parthiban Marimuthu, Christa E. Müller, Steven E. Mansoor

**Affiliations:** ^a^PharmaCenter Bonn and Pharmaceutical Institute, Department of Pharmaceutical & Medicinal Chemistry, University of Bonn, Bonn 53121, Germany; ^b^Research Training Group 2873, University of Bonn, Bonn 53121, Germany; ^c^Department of Chemical Physiology & Biochemistry, Oregon Health & Science University, Portland, OR 97239; ^d^Pharmaceutical Science Laboratory (Pharmacy) and Structural Bioinformatics Laboratory (Biochemistry), Faculty of Science and Engineering (FNT), Åbo Akademi University, Turku FI-20520, Finland; ^e^Division of Cardiovascular Medicine, Knight Cardiovascular Institute, Oregon Health & Science University, Portland, OR 97239

**Keywords:** ATP, purinergic P2X2 receptor, ligand-gated ion channels, cryo-EM, molecular dynamics (MD) simulations

## Abstract

The P2X2 receptor (P2X2R) is a slowly desensitizing adenosine triphosphate (ATP)-gated ion channel that is highly expressed in the cochlea. When mutated, the P2X2R exacerbates age- and noise-related hearing loss, but selective modulators of the receptor are lacking, and the molecular basis of activation and desensitization remains poorly understood. Here, we determine high-resolution cryoelectron microscopy structures of the full-length wild-type human P2X2R in an apo closed state and two distinct ATP-bound desensitized states. In the apo closed state structure, we observe features unique to the P2X2R and locate disease mutations within or near the transmembrane domain. In addition, our ATP-bound structures show how free anionic ATP forms subtype-specific interactions with the orthosteric binding site. We identify and characterize two different ATP-bound desensitized state structures, one similar to published models for other P2XR subtypes, and a second alternate conformation not previously observed. A loop adjacent to the orthosteric binding site between these two ATP-bound desensitized state structures undergoes significant conformational changes. These movements are supported by multireplicate, microsecond-scale molecular dynamics simulation studies and suggest a path by which ATP could enter or leave the orthosteric pocket. Together, our results provide structural insights into the P2X2R, facilitating structure-based drug development for this therapeutically important target.

## Significance

The P2X2 receptor (P2X2R) is an adenosine triphosphate (ATP)-gated ion channel with few small-molecule modulators that exhibit poor pharmacological properties. It is known to be involved in the auditory system and has been proposed as a drug target for age- and noise-related hearing loss. We provide high-resolution cryoelectron microscopy structures of the full-length wild-type human P2X2R in an apo closed state and two ATP-bound desensitized state conformations. These data provide structural details of the P2X2R, revealing key subtype-specific features and a platform to facilitate rational structure-based drug design for this therapeutically relevant receptor.

The nucleotide adenosine triphosphate (ATP) was first described as an extracellular neurotransmitter in 1972 (1). Since then, it has become apparent that extracellular ATP is an essential signaling molecule, interacting with two different families of purinergic receptors (2–4). The seven P2X receptor subtypes (P2X1R-P2X7R) constitute ATP-gated ion channels whereas the eight P2Y receptor subtypes are nucleotide-activated G protein–coupled receptors (5, 6). P2X receptors (P2XRs) have been extensively studied over the past decades for their significant roles in inflammation and pain (5, 7). From a pharmaceutical research perspective, major advancements have been made in drug development targeting P2X3R and P2X7R. Indeed, the P2X3R-selective antagonist gefapixant has recently been approved for the treatment of inflammation-mediated chronic cough (8, 9). Moreover, subtype-selective antagonists have been developed for the P2X7R, several of which are in advanced preclinical or clinical trials (10). In contrast, much less is known about the molecular pharmacology of the P2X2R, for which only few modulators are known, that exhibit unsatisfactory physicochemical properties, poor potency, and/or lack of selectivity (5, 11).

The P2X2R is predominantly expressed in the central nervous system and on peripheral postsynaptic neurons (5, 12). For example, P2X2Rs in the cochlea play an important role in adaptation to loud noise and protection from overstimulation (5, 13–19), and previous studies have identified clinically relevant disease mutations in the P2X2R that exacerbate hearing loss (15, 20–24). Harnessing the protective effect of P2X2Rs could be a valuable strategy for the treatment of hearing loss, which is currently the third-leading chronic health condition in the United States and predicted to double by 2060 due to the aging population (25–27). The P2X2R has also been implicated in other pathophysiological processes. For example, elevated extracellular ATP levels following intestinal surgery can induce P2X2R-mediated enteric inflammation and gliosis, causing impairment of ileal motility (28). Encouragingly, P2X2R blockade reduced ileal inflammation (28), raising hope that the P2X2R could be a drug target for inflammatory diseases in addition to noise- and age-related hearing loss (5, 23).

The lack of pharmacological advancements for the P2X2R is in part due to the absence of structural information for this P2XR subtype. To date, high-resolution structures have been reported for P2X1R, P2X3R, P2X4R, and P2X7R, revealing their distinctive trimeric architecture and ATP-binding domains (29–44). However, many of these studies used either truncated, mutated, and/or nonhuman constructs to enable structural determination by X-ray crystallography or cryogenic electron microscopy (cryo-EM). In each case where a full-length wild-type construct was used, substantial information was gained correlating molecular structure to the physiological function of the corresponding P2XR subtype (36, 38–41, 44). Moreover, several subtypes of this functionally diverse family, including the homotrimeric P2X2R, P2X5R, and P2X6R as well as any heterotrimeric P2XR, have yet to be characterized at a structural level, which is essential for defining subtype-selectivity of agonists and antagonists as well as mechanisms of activation and gating. Indeed, it is generally accepted that experimentally derived structures are nearly indispensable for advancing drug discovery efforts (45, 46).

To investigate the structural basis of P2X2R pharmacology and identify subtype-specific molecular features, we used cryo-EM to characterize the human P2X2R (hP2X2R) expressed in mammalian cells. Our apo closed state structure reveals the composition of the orthosteric binding pocket, the architecture of the closed pore, and the location of known disease-causing mutations. In addition, two distinct structures in the ATP-bound desensitized state, supported by molecular dynamics (MD) simulation studies, define unique interactions between ATP and the orthosteric binding site as well as structural movements that could underlie the entry or exit of ATP into or from the orthosteric pocket, respectively. By leveraging comparisons with previously published P2X1R, P2X3R, P2X4R, and P2X7R structures, we highlight unique characteristics of the P2X2R that may play a role in subtype-specific physiology and pharmacology. These insights will provide a starting point for structure-based drug design for a clinically relevant receptor.

## Results

### The Apo Closed State Conformation of hP2X2R Has Unique Structural Features.

**Fig. 1.**
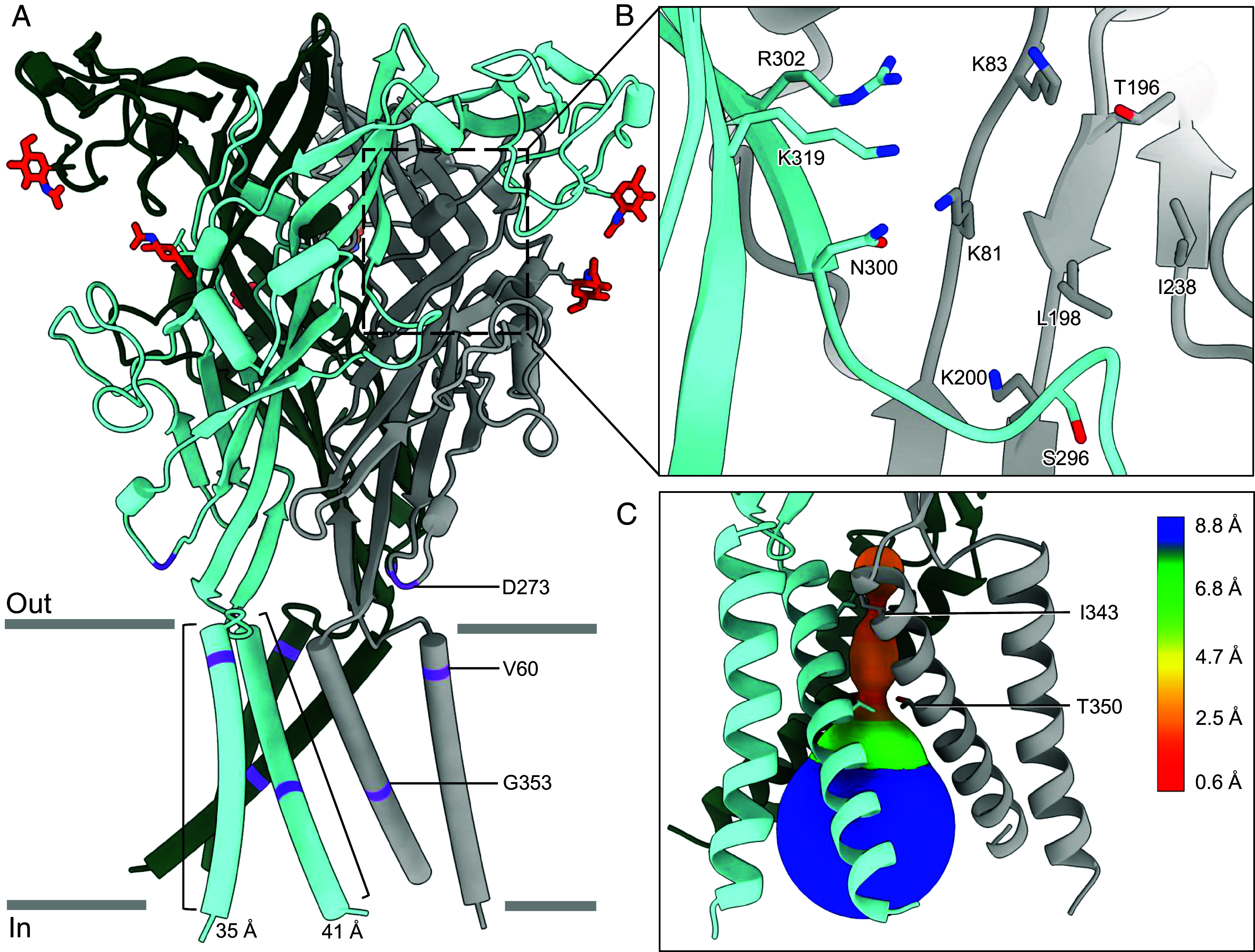
Structure and pore architecture of hP2X2R in the apo closed state conformation. (*A*) Ribbon representation of hP2X2R in the apo closed state shown in a side view, parallel to the cell membrane. Each protomer is colored differently (light blue, green, and gray). Glycosylation is colored by atom: carbon in orange, nitrogen in blue, and oxygen in red. The locations of reported mutations causing hearing loss (V60L, D273Y, and G353R) are labeled and indicated in purple on the structure. Both TM helices were visualized with nearly seven turns per helix each and exhibit a length of 35 Å and 41 Å for TM1 and TM2, respectively. The length was measured with ChimeraX (47, 48). The location of the empty orthosteric ATP-binding site is indicated by the dotted black box. (*B*) Magnified view of the empty orthosteric binding pocket, highlighting the residues that interact with ATP. The surface-accessible volume of the orthosteric binding site is ~2,700 ± 400 Å^3^ as calculated with the program Fpocket (49). The side chains shown are colored by atom: nitrogen in blue and oxygen in red. (*C*) The ion permeation pathway of hP2X2R in the apo closed state reveals that the constriction gate of the closed pore at its narrowest point is 0.6 Å, created by the symmetry-related side chains of residue I343 on TM2. For the pore size plot, different colors represent different radii, as generated by the program MOLE2.5 (50).

To investigate the subtype-specific features of P2X2Rs, we used cryo-EM to determine the structure of full-length wild-type hP2X2R in an unliganded conformation (Fig. 1*A* and *SI Appendix*, Figs. S1 and S2*A* and Table S1). With a constricted pore and an absence of ATP in the orthosteric binding site, this structure represents an apo closed state —a key conformation for defining unliganded pockets and understanding the molecular changes resulting from subsequent agonist binding (Fig. 1*B*). We refined this cryo-EM reconstruction to 2.7 Å, allowing detailed structural visualization of this P2XR subtype (*SI Appendix*, Fig. S2*A*).

In line with other structurally determined P2XR subtypes, the apo closed state conformation of hP2X2R reveals that the receptor is a trimeric protein with pruned RMSDs of 0.9 Å, 0.9 Å, 1.0 Å, and 1.2 Å in comparison to human P2X1R (hP2X1R), human P2X3R (hP2X3R), human P2X4R (hP2X4R), and rat P2X7R (rP2X7R), respectively (PDB IDs: 9C2A, 5SVJ, 9BQH, 6U9V, respectively) (35, 36, 38, 40). Each P2X2 protomer is composed of intracellular N and C termini and two transmembrane (TM) helices (outer TM1 and pore-lining TM2) connected by a voluminous extracellular domain. Three P2X2 protomers intertwine to form the trimeric receptor, with each subunit adopting a dolphin-like shape, as first established for the zebrafish P2X4R (zfP2X4R) (*SI Appendix*, Fig. S3) (37). The upper and lower bodies, the dorsal fin, the left and right flippers, and the head collectively form the extracellular domain whereas the fluke forms the transmembrane domain (TMD) (*SI Appendix*, Fig. S3).

In the apo state conformation, the three TM2 helices are arranged in a “teepee” conformation to form the closed pore (Fig. 1 *A* and *C*) (35, 51). In contrast to the structures of zfP2X4R, each TM helix in hP2X2R is fully visualized (TM1 and TM2 helices are 35 Å and 41 Å in length, respectively) with lengths similar to the TM helices of hP2X4R (*SI Appendix*, Fig. S4 *A* and B) (37, 40, 42). Further, the structure of full-length wild-type hP2X2R in the apo closed state establishes the correct helical pitch of each TM helix, not present in either truncated structures of other P2XR subtypes or the AlphaFold 3 predicted model of the hP2X2R (*SI Appendix*, Figs. S4 *C* and D and S5) (52). Despite using a full-length wild-type hP2X2R construct, the cytoplasmic domain, including the cytoplasmic cap and any additional cytoplasmic residues, were not present in the cryo-EM reconstructions, likely due to high conformational flexibility of the reconstituted receptor (35, 36, 40, 51).

There are several subtype-specific features of hP2X2R worth highlighting. For example, in our reconstructions of the receptor, expressed in human embryonic kidney (HEK) cells, there is strong cryo-EM density for glycosylation of two residues per protomer (N133 and N194) and weak cryo-EM density for the third (N310) out of three consensus motifs for N-linked glycosylation. Thus, only two glycosylations per protomer are shown in the final models (Fig. 1*A*). In contrast, three, three, six, and five glycosylated asparagine residues per protomer were observed in the structures of hP2X1R, hP2X3R, hP2X4R, and rP2X7R, respectively (35, 36, 38, 40). N-linked glycosylation is essential for the surface presentation of the hP2X2R (53, 54). In addition, the surface-accessible volume of the orthosteric binding pocket in the hP2X2R is ~2,700 ± 400 Å^3^ as calculated by the program Fpocket (49), similar to that of the hP2X4R (~2,700 ± 19 Å^3^) and the rP2X7R (~2,700 ± 35 Å^3^), but strikingly smaller than the binding pocket volumes of the hP2X1R (~3,300 ± 200 Å^3^) and the hP2X3R (~5,100 ± 20 Å^3^) (*SI Appendix*, Fig. S6) (29, 35, 38–40). These differences appear to be a result of distinct conformations of the dorsal fin and left flipper domains, which narrow the ATP-binding pocket in the apo closed state conformation of some P2XR subtypes including the hP2X2R (*SI Appendix*, Fig. S3).

The recently characterized allosteric antagonist-binding pocket of the hP2X4R has a distinct residue composition compared to the same site in the hP2X2R, identifying a feature that can be leveraged for subtype-specific ligand development (40). For example, the small-molecule BAY-1797 potently inhibits the hP2X4R and is selective over other P2XR subtypes (5, 55). Aligning the cryo-EM structure of hP2X4R in the BAY-1797-bound inhibited state (PDB ID: 9BQI) with the apo closed state structure of hP2X2R reveals that several residues forming the allosteric pocket of the hP2X4R are different between these two subtypes (*SI Appendix*, Fig. S7) (40). For example, E97, K100, and P102 in hP2X2R correspond to D88, I91, and A93 in hP2X4R, respectively, both altering the size and charge of the corresponding allosteric site in hP2X2R (*SI Appendix*, Fig. S7). It is known that mutation of residues D88 and I91 in hP2X4R dramatically reduces the hP2X4R-antagonistic potency of BAY-1797 (30). Functional validation of this hypothesis still needs to be performed. However, comparison of the subtype-specific residues within the analogous pocket of the hP2X2R provides an important basis for subtype-specific structure-based drug design.

The locations of the hP2X2R hearing loss mutations were previously predicted using the known structure of the hP2X3R (sequence identity 37.2%) (15). In agreement with these predictions, we observed V60 at the extracellular end of TM1 interacting with I339 of the neighboring protomer, D273 on a β-turn in the lower body at an interface with the TMD, and G353 on TM2, directly next to one of the pore-lining residues V354 (Fig. 1*A* and *SI Appendix*, Fig. S8). These observations confirm the location of hearing loss mutations within or near the TMD, verifying the quality of the previous prediction (15).

### ATP-Bound hP2X2R Enters a Canonical Desensitized State Conformation.

**Fig. 2.**
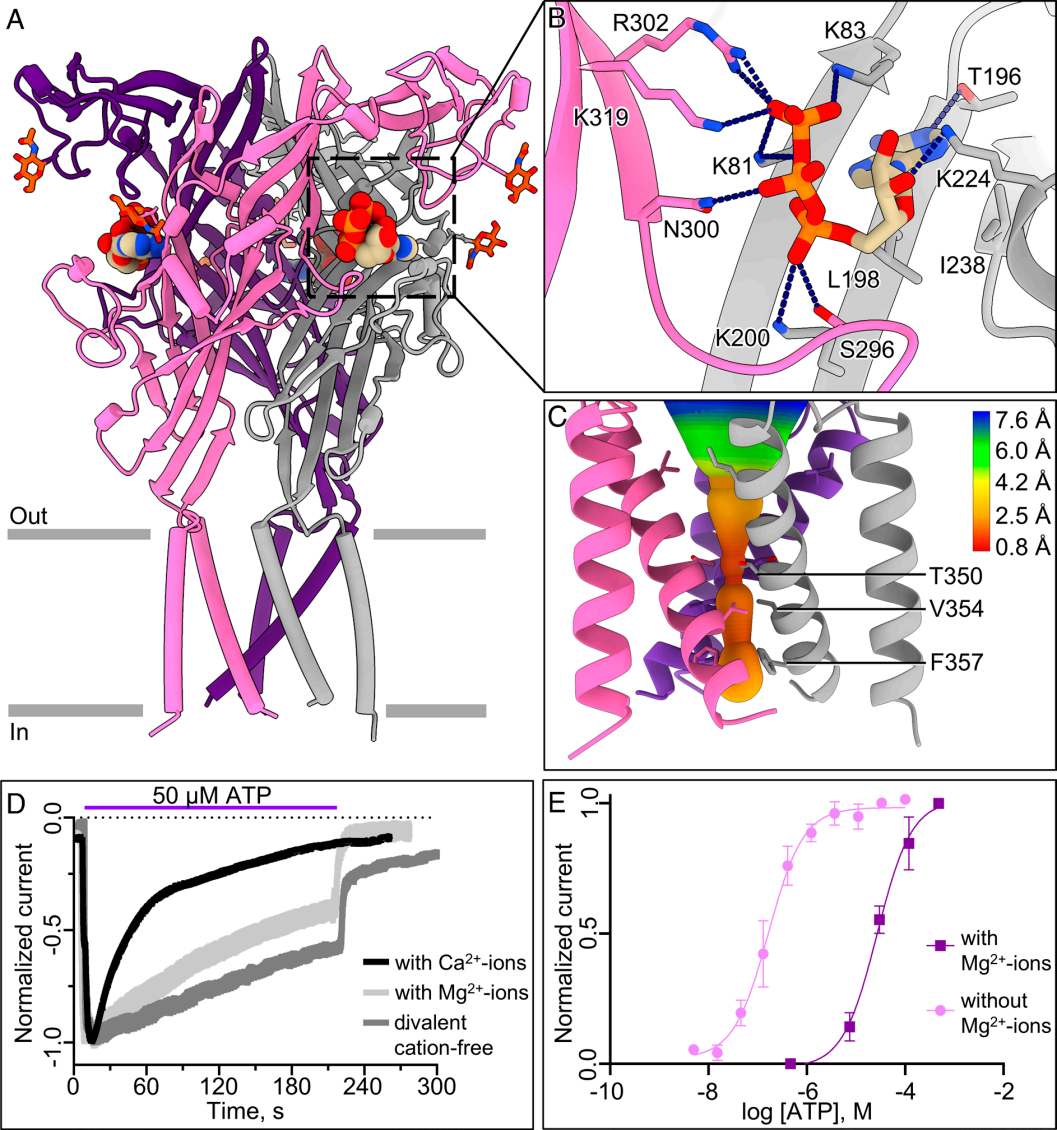
Structure and pore architecture of hP2X2R in the canonical ATP-bound desensitized state conformation and ATP-activation studies in *Xenopus* oocytes. (*A*) Ribbon representation of hP2X2R in the canonical ATP-bound desensitized state, highlighting one of the three occupied orthosteric ATP-binding sites (outlined in a black dotted box). Glycosylation is colored by atom: carbon in orange, nitrogen in blue, and oxygen in red. Each protomer of the receptor is colored differently (pink, purple, and gray). (*B*) Magnified view of ATP bound in the orthosteric pocket, highlighting the key interactions (interaction distances of hydrogen bonds and salt bridges: K81—2.9 Å and 3.1 Å, K83—3.4 Å, T196—3.3 Å, K200—4.0 Å, K224—2.8 Å, S296—2.8 Å, N300—3.0 Å, R302—2.9 Å and 2.9 Å, K319—3.8 Å). The side chains shown and the heteroatoms of ATP are colored by atom: nitrogen in blue, oxygen in red, and phosphorus in orange. The carbon atoms of ATP are shown in tan. All distances were measured in ChimeraX (47, 48). (*C*) The ion permeation pathway of hP2X2R in the ATP-bound desensitized state reveals that the constriction gate of the closed pore at its narrowest point is 0.8 Å, created by the symmetry-related side chains of residue V354 on TM2. For the pore size plot, different colors represent different radii, as generated by the program MOLE2.5 (50). (*D*) Representative desensitization profiles of the hP2X2R upon activation with 50 μM ATP revealing different rates of desensitization in recording buffer containing 5 mM Ca^2+^-ions, 5 mM Mg^2+^-ions or without divalent cations. (*E*) The concentration–response curve (EC_50_) for wild-type hP2X2R activated by ATP in divalent cation-free conditions is measured to be 0.156 ± 0.096 μM by two electrode voltage clamp (TEVC) in *Xenopus* oocytes. In the presence of Mg^2+^-ions in the recording buffer, the EC_50_ is determined to be 28.6 ± 8.1 μM. The y-axis describes currents normalized to the largest current evoked by the maximum concentration of ATP applied to each oocyte (100 μM for buffer without Mg^2+^-ions and 1,920 μM for buffer with Mg^2+^-ions). Each reported EC_50_ with error bars represents the mean ± SD across triplicate experiments.

Having established the structural features of hP2X2R in the apo closed state, we next investigated the molecular basis of its activation by determining the structure of full-length wild-type hP2X2R bound to ATP (Fig. 2 *A*–*C* and *SI Appendix*, Figs. S1, S2 *B* and C, S9, and S10 and Table S1). Prior to structural determination, we carried out two-electrode voltage clamp (TEVC) recordings of full-length wild-type hP2X2R expressed in *Xenopus* oocytes, the same construct as used for final structure determination. In agreement with previous studies, hP2X2R channels opened quickly upon application of ATP and desensitized slowly (Fig. 2*D* and *SI Appendix*, Fig. S11) (56–58). At a physiological pH value of 7.4, the ion channel responded to ATP with an EC_50_ of 0.156 ± 0.096 μM in the absence, and of 28.6 ± 8.1 μM in the presence of Mg^2+^-ions (5 mM) in the recording buffer (Fig. 2*E*) (57, 59). Consistent with previous studies, the effects of Ca^2+^- and Mg^2+^-ions on desensitization kinetics, as well as their negative modulatory activity on the P2X2R, were confirmed here (Fig. 2 *D* and *E*) (56, 60, 61). It is important to consider these modulatory effects in tissues such as the cochlea where P2X2R is highly expressed on cells exposed to body fluids with varying concentrations of divalent cations under physiological and pathophysiological conditions (16, 62). Studies show that the level of divalent cations plays a role in adjusting the response of the cochlea to sound, and elevated Ca^2+^-levels have been reported as a consequence of gentamicin-induced ototoxicity (62, 63).

Using cryo-EM we determined two distinct structures (obtained from two different purification approaches) with ATP bound in the orthosteric binding sites and a uniquely constricted pore, consistent with desensitized state conformations (Fig. 2 *A*–*C* and *SI Appendix*, Fig. S1) (51). Both desensitized state structures, which we refer to as conformation I and conformation II, were determined at high resolution (2.5 Å, and 2.6 Å, respectively) (*SI Appendix*, Fig. S2 *B* and C and Table S1). They are nearly identical (pruned RMSD of 0.5 Å), except for a key difference in the left flipper domain adjacent to the orthosteric binding pocket (discussed below) and TM1 (*SI Appendix*, Fig. S12). While both structures are similar to the ATP-bound desensitized state structure of hP2X3R (RMSDs of 0.7 Å and 0.8 Å, respectively) (PDB ID: 5SVL) (35), the left flipper in conformation I mirrors the left flipper in the desensitized state conformations of the hP2X1R, hP2X3R, and hP2X4R (*SI Appendix*, Fig. S3). Thus, we refer to conformation I as the canonical desensitized state structure and establish conformation II as an alternate ATP-bound desensitized state structure.

In both desensitized state structures, the three symmetrical ATP-binding sites are positioned at the interfaces of adjacent P2X2 protomers (Fig. 2 *A* and *B*). The interfacial pockets are located ~58 Å away from the pore, each surrounded by residues in the head, left flipper, and upper body of one subunit, and the dorsal fin and lower body of the neighboring subunit (*SI Appendix*, Fig. S3). Most of the residues in the orthosteric binding pocket are positively charged and hydrophilic, ideal for interacting with negatively charged, highly polar ATP molecules. Indeed, anionic ATP binds in a U-shaped conformation due to multiple ionic interactions and hydrogen bonds that have been observed in other P2XR subtypes (Fig. 2*B* and *SI Appendix*, Figs. S9 and S10) (29, 31, 35–38, 40–42). The triphosphate tail forms ionic interactions with K81, K83, K200, R302, and K319 and a hydrogen bond with N300. The adenine core of ATP interacts with the lipophilic residues K83 and T196, and the hydrophobic residues L198 and I238. Further, the 3’-hydroxy-group of the ribose moiety interacts with K224 via hydrogen bonding, an interaction unique to hP2X2R (Fig. 2*B*). Finally, present only in conformation I, a hydrogen bond is formed between S296 and the α-phosphate of ATP. Thus, our data show that following channel activation by ATP, hP2X2Rs enter a canonical desensitized state (conformation I) but with features unique to this receptor subtype.

Unlike previously observed for hP2X1R, hP2X3R, and hP2X4R, the absence of cryo-EM density for any ion coordinating ATP in the pocket suggests that free anionic ATP rather than an ATP–cation complex is the physiological ligand for P2X2Rs (35, 38, 40, 41, 43, 64). By closely examining the structures of P2XRs bound to MgATP^2−^ (hP2X1R, hP2X3R, and hP2X4R with PDB IDs: 9C2B, 6AH5, 9C48, respectively), we identified a striking difference between previous structures and the hP2X2R structure in the transition area of the head and right flipper domains (residues E179–L188) (*SI Appendix*, Figs. S3 and S13) (38, 40, 43). At the position of the aspartate residue that coordinates the Mg^2+^-ion in hP2X1R (D170), hP2X3R (D158), and hP2X4R (D170), a glycine residue (G181) is present in the hP2X2R (*SI Appendix*, Fig. S13*A*). It is well known that glycine residues can act as secondary structure breakers, and indeed, we find the “Mg^2+^-coordinating region” observed in other P2XR subtypes to be rotated away from the orthosteric binding pocket in hP2X2R (*SI Appendix*, Fig. S13 *B* and C) (65, 66). Although an aspartate (D180) is present in close proximity, the distance (>3.6 Å) is too far to allow for coordination with MgATP^2−^ (*SI Appendix*, Fig. S13*D*). We employed TEVC to evaluate whether the apparent affinity of hP2X2R for MgATP^2−^ can be artificially increased by mutating glycine to aspartate (G181D) and alanine to threonine (A182T), in analogy to the residues present in hP2X3R (D158 and T159) and hP2X4R (D170 and T171). Surprisingly, introducing these mutations into the hP2X2R did not significantly impact the apparent affinity of the mutated receptors for ATP^4−^ or MgATP^2−^ (*SI Appendix*, Fig. S14).

### ATP-Bound hP2X2R Can Enter an Alternate Desensitized State Conformation.

**Fig. 3.**
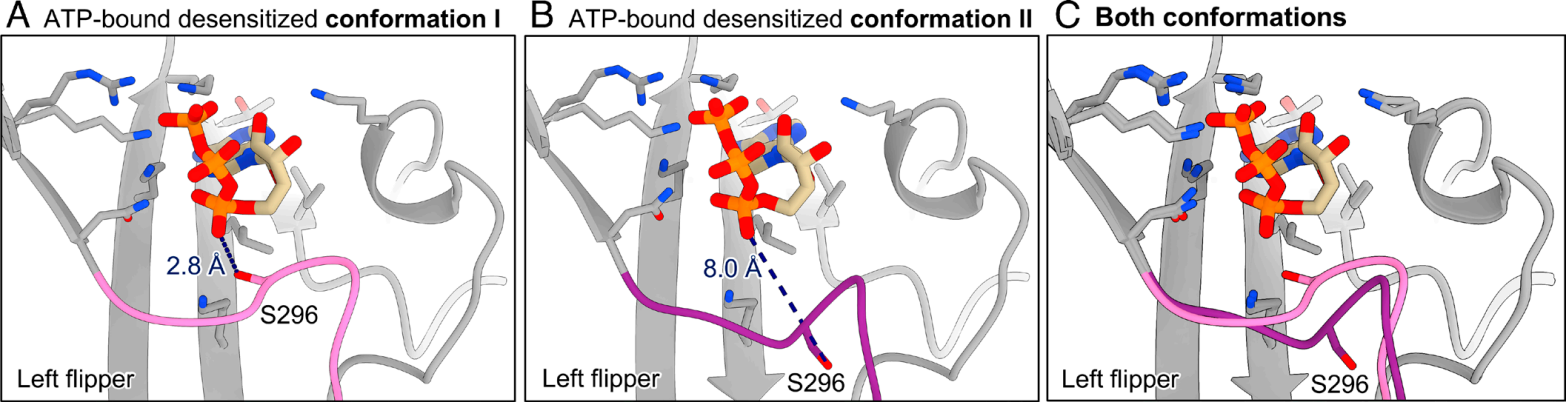
The left flipper can occupy two conformations in the ATP-bound desensitized state of hP2X2R. The left flipper found in the canonical ATP-bound desensitized state (pink) has a distinctly different conformation compared to the alternate ATP-bound desensitized state conformation (purple). This conformational change results in differences in the interaction of S296 and the α-phosphate of ATP. The side chains shown and the heteroatoms of ATP are colored by atom: nitrogen in blue, oxygen in red, and phosphorus in orange. The carbon atoms of ATP are shown in tan. (*A*) The canonical ATP-bound desensitized state (conformation I) contains a hydrogen bond between S296 and the α-phosphate of ATP [distance: 2.8 Å as measured in ChimeraX (47, 48)]. (*B*) The left flipper in an alternate ATP-bound desensitized state (conformation II) has a different structure, with S296 pointing away from ATP, unable to make the hydrogen bond present in conformation I [distance: 8.0 Å as measured in ChimeraX (47, 48)]. (*C*) Alignment of conformation I and conformation II highlighting the significant structural difference of the left flipper domain between the two models. Compared to conformation I, S296 in conformation II is flipped away from ATP.

We observed one significant difference between the ATP-bound desensitized state structures that delineate two distinct conformations. In conformation II, the left flipper (residues 288–299) adopts an alternate pose compared to conformation I, abrogating an interaction with ATP (Fig. 3 and *SI Appendix*, Figs. S3, S9, S12, and S15). In conformation I, S296 interacts with the α-phosphate of ATP via a hydrogen bond at a distance of 2.8 Å (Fig. 3*A* and *SI Appendix*, Fig. S15*A*). In contrast, in conformation II, the loop containing S296 is flipped away from the orthosteric pocket, resulting in S296 pointing in the opposite direction, now at a distance of 8.0 Å from the α-phosphate of ATP (Fig. 3*B* and *SI Appendix*, Fig. S15*B*). As a result of this alternate conformation, there is one less interaction with ATP and a larger opening to the ATP-binding pocket (Fig. 3).

We performed multireplicate, microsecond-scale MD simulations (1 µs) to probe the three different conformational states of the hP2X2R that we identified by cryo-EM. The simulations using our apo closed state structure reveal that the left flipper is a conformationally flexible region, with an average fluctuation of 2.78 ± 0.81 Å calculated as RMS fluctuation (RMSF) across the three chains and all replicates (*SI Appendix*, Fig. S16 and Movie S1).

Next, we performed similar MD simulations starting from conformation I, in which ATP is tightly bound inside the orthosteric pocket, and the loop of the left flipper adopts a “closed pocket” conformation. Following protein preparation for MD simulations, S296 directly interacts with the α-phosphate of ATP through a hydrogen bond at an initial distance of 1.6 Å. Across five replicate simulations, the left flipper continued to show flexibility (*SI Appendix*, Figs. S17 and S18). Specifically, the side chain of S296 moved away from the α-phosphate of ATP, breaking the hydrogen bond to reach distances of over 10 Å, but in some simulations, this interaction was reestablished. In more than 20% of the simulation time, the distance between S296 and the α-phosphate of ATP is less than 4.0 Å (potential hydrogen bond distance) (*SI Appendix*, Figs. S19 *A–C* and S20 and Movie S2).

Finally, when conformation II was used for MD simulations, the left flipper starts in an “open pocket” conformation, with an initial distance between S296 and the α-phosphate of ATP of 8.0 Å. The left flipper of conformation II was also mobile during the simulations moving between distances above and below 4.0 Å for the aforementioned interaction (*SI Appendix*, Figs. S17, S18, S19 *D–F,* and S21 and Movie S3). Supported by the MD simulations, the hP2X2R in the ATP-bound desensitized state can adopt a range of conformations including both conformation I and conformation II (*SI Appendix*, Figs. S19–S23 and Movies S2 and S3).

### Apo Closed and ATP-Bound Desensitized State Conformations in hP2X2R Have Different Gates.

**Fig. 4.**
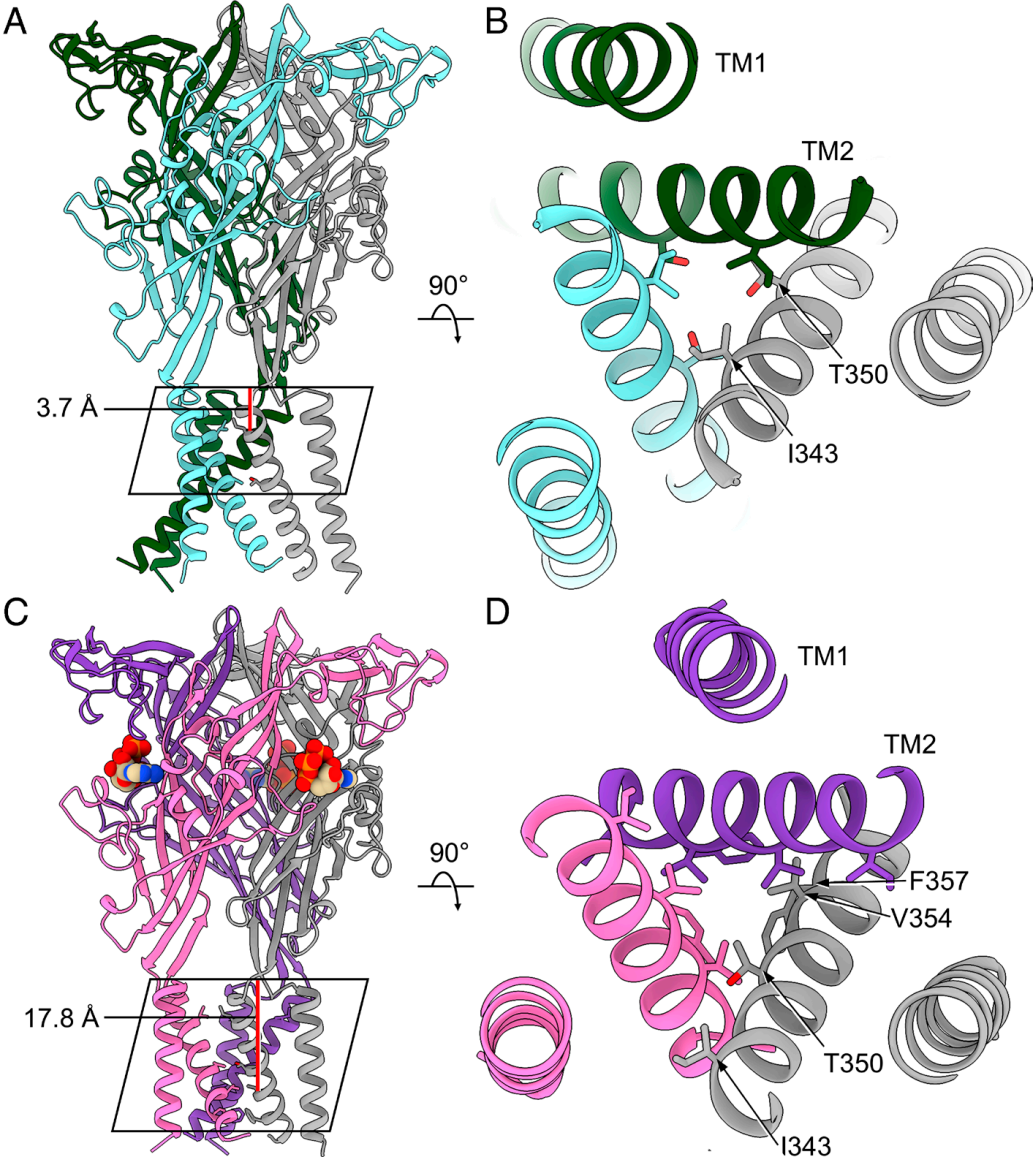
Comparison of the pore for hP2X2R in the apo closed state conformation to the pore in the canonical ATP-bound desensitized state conformation. Different residues make up the gate in the apo closed and ATP-bound desensitized state conformations. Each protomer is depicted in a different shade. The side chains shown and the heteroatoms of ATP are colored by atom: nitrogen in blue, oxygen in red, and phosphorus in orange. The carbon atoms of ATP are shown in tan. (*A*) Overall topology of the apo closed state. The primary constriction gate (I343) is only 3.7 Å deep into the plasma membrane. Due to the shallow depth of the gate (indicated with a red line), the overall shape of the transmembrane domain resembles a teepee. The depth of the gate was measured using the distance between centroids generated from Cα L338 to Cα I343, from each protomer in ChimeraX (47, 48). (*B*) Top–down view of the pore in the apo closed state. Amino acid residues (I343 and T350) involved in the pore constrictions are labeled and shown as stick models. (*C*) Overall topology of the canonical ATP-bound desensitized state. The primary constriction gate (V354) in the ATP-bound desensitized state structure is 17.8 Å deep into the plasma membrane, a full 14.1 Å deeper than the constriction gate of the apo closed state. Due to the deeper gate (indicated with a red line), the overall shape of the transmembrane domain in the ATP-bound desensitized state resembles a cone. The depth of the gate was measured using the distance between centroids generated from Cα L338 to Cα V354, from each protomer in ChimeraX (47, 48). (*D*) Top–down view of the pore in the ATP-bound desensitized state. Amino acid residues (T350, V354, and F357) involved in the pore constriction are labeled and shown as stick models. I343 is shown additionally to visualize the different position of this residue compared to the apo closed state.

Despite similar arrangements of the extracellular domains in the apo closed and ATP-bound desensitized states, we observed key differences in the TMDs framing the ion-permeation pathway (Fig. 4). In each hP2X2R structure, the pore is lined by TM2 helices from each protomer, which influence the conduction pathway for ion permeation. In the apo closed state, the three symmetry-related side chains of I343 form the primary constriction gate (0.6 Å radius) (Figs. 1*C* and 4 *A* and *B* and Table 1). In addition, another constriction formed by the symmetry-related side chains of T350 comprises a second, more cytoplasmic gate (1.1 Å radius) (Figs. 1*C* and 4*B*). The primary constriction gate in the apo closed state is only 3.7 Å deep into the plasma membrane, as measured from the start of TM2 (Cα of L338 to Cα of I343), and thus the TMD forms a teepee shape (Fig. 4*A* and Table 1) (51).

**Table 1.** Comparison of features for the different gates of hP2X1R, hP2X2R, hP2X3R, hP2X4R, and rP2X7R in the apo closed and ATP-bound desensitized state structures^*^

	Closed apo		ATP-bound desensitized	
	Pore radius	Gate depth	Pore radius	Gate depth
hP2X1R	S337 (radius 0.4 Å)	7.9 Å deep:	V334 (radius 0.6 Å)	16.9 Å deep:
		Cα S337–Cα I328		Cα I328–Cα V344
hP2X2R	I343 (radius 0.6 Å)	3.7 Å deep:	T350 (radius 0.9 Å)	17.8 Å deep:
	T350 (radius 1.1 Å)	Cα I343–Cα L338	V354 (radius 0.8 Å)	L338–Cα V354
			F357 (radius 0.9 Å)	
hP2X3R	I323 (radius 0.4 Å)	3.9 Å deep:	V334 (radius 1.5 Å)	15.9 Å deep:
		Cα I323–Cα I318		Cα I338–Cα V354
hP2X4R	I337 (radius 1.0 Å)	12.9 Å deep:	M348 (radius 0.1 Å)	18.2 Å deep:
	A344 (radius 0.4 Å)	Cα A344–Cα I332		Cα I338–Cα M354
rP2X7R	S339 (radius 0.1 Å)	13.6 deep:	Does not desensitize	
	S342 (radius 0.1 Å)	Cα S342–Cα I330		

^*^The pore radius was calculated using the program HOLE (67). The depth for each gate was measured using the distance between centroids generated from two sets of Cα atoms, one from the start of TM2 and the other from the gate forming residue, from each protomer in ChimeraX (47, 48). PDB IDs: 9C2A (hP2X1R, apo closed) (38), 9C2B (hP2X1R, ATP-desensitized) (38), 5SVJ (hP2X3R, apo closed) (35), 5SVL (hP2X3R, ATP-desensitized) (35), 9BQH (hP2X4R, apo closed) (40), 9C48 (hP2X4R, ATP-desensitized) (40), 8TR5 (rP2X7R, apo closed) (39). h—human, r—rat.

The pore architecture of the hP2X2R in the apo closed state has intriguing properties. The depth of the constriction gate of the hP2X2R (3.7 Å, Cα of L338 to Cα of I343) is very similar to that of the hP2X3R (3.9 Å, Cα of I318 to Cα of I323) (Table 1) (35). In contrast, the constriction gates of hP2X1R, hP2X4R, and rP2X7R are deeper into the membrane bilayer compared to the hP2X2R by ~4 Å (7.9 Å, Cα of I328 to Cα of S337), ~8 Å (12.9 Å, Cα of I332 to Cα of A344), and ~9 Å (13.6 Å, Cα of I330 to Cα of S342), respectively (Table 1) (36, 38, 40). Because of the analogous architecture of the extracellular domain and the pore, the hP2X3R is likely the best-suited P2XR subtype among the structurally determined P2XRs to form functional heterotrimers with the hP2X2R (5).

The transition to the desensitized state, described by the helical recoil model of P2XR desensitization, causes movements of the TMD that set the gate in the ATP-bound desensitized state (51). The pore of hP2X2R in the ATP-bound desensitized state is constricted by three sets of residues on TM2 (Figs. 2*C* and 4 *C* and *D* and Table 1). An initial constriction is formed by the side chains of residue T350 from each protomer (pore radius of 0.9 Å) (Figs. 2*C* and 4 *C* and *D*). One helical turn more cytoplasmic, the narrowest part of the gate is formed by the side chains of residue V354 from each protomer and constricts the pore to a radius of 0.8 Å, thus defined as the primary constriction gate (Figs. 2*C* and 4*C*). The final residue that contributes to the constriction is yet another helical turn more cytoplasmic, formed by the side chains of residue F357 from each protomer (pore radius of 0.9 Å) (Figs. 2*C* and 4*D*). The primary gate in the ATP-bound desensitized state of hP2X2R is now 17.8 Å deep into the plasma membrane, as measured from the start of TM2 (Cα of L338 to Cα of V354) (Fig. 4*C*). As a result of the deeper gate compared to the apo closed state, the TMD architecture in the ATP-bound desensitized state now adopts a “cone” shape (Fig. 4*C*) (51).

Across ATP-bound desensitized state structures of P2XRs, the primary constriction gate in hP2X2R (V354) is at the same position as the constriction gate of hP2X1R (V334), hP2X3R (V334), and hP2X4R (M348) (Table 1) (35, 38, 40). In addition, the constriction gates of hP2X1R, hP2X2R, hP2X3R, and hP2X4R are all similarly deep within the membrane bilayer with distances of 16.9 Å (Cα of I328 to Cα of V344), 17.8 Å (Cα of L338 to Cα of V354), 15.9 Å (Cα of I318 to Cα of V334), and 18.2 Å (Cα of I332 to Cα of M348), respectively (35, 38, 40). In contrast to the relatively discrete constriction gates found in hP2X1R, hP2X3R, and hP2X4R that are formed at one single major point of constriction, the pore in hP2X2R uniquely constricts across three sets of residues over two full helical turns of TM2, extending between residues T350 and F357 (Figs. 2*C* and 4*D* and Table 1). The longer, more constricted pore of hP2X2R appears to be a unique feature of this P2XR subtype.

Altogether, in both the apo closed and ATP-bound desensitized states of hP2X2R, the primary constrictions are too narrow to allow Na^+^-ions to pass through the pore, indicating that these constrictions form gates that render the channel nonconducting (68, 69).

## Discussion

Our high-resolution structures of full-length wild-type hP2X2R in an apo closed and two distinct ATP-bound desensitized state conformations provide insight into the subtype-specific structural features of this important receptor. The apo closed state structure reveals the composition of the orthosteric ATP-binding pocket, the architecture of the closed pore, and the location of clinically relevant disease mutations. The structure of the canonical ATP-bound desensitized state (conformation I) identifies subtype-specific interactions with ATP in the orthosteric pocket and a second ATP-bound desensitized state structure (conformation II) reveals that movement of the left flipper is potentially a transition step that facilitates the entry or the departure of ATP in or out of the pocket, respectively. Thus, the open pocket conformation II might represent a transition state for the association or dissociation of ATP to and from P2X2R (*SI Appendix*, Figs. S24 and S25) (70).

The ATP-bound desensitized state conformation I was obtained from a purification strategy that closely followed a protocol previously established for other P2XR subtypes (35, 38, 40). This structure was determined without the addition of ATP to the purified protein sample, leading to the conclusion that intracellular ATP released during cell lysis binds to the receptor. In a subsequent attempt to capture an apo state conformation, we added apyrase prior to cell lysis to hydrolyze endogenous ATP. Ca^2+^-ions were applied as a cofactor to activate apyrase and to negatively modulate P2X2R activation (56, 60, 61). During cryo-EM data processing from the second purification, we were able to classify two distinct conformational states; the apo closed state conformation in addition to the alternate ATP-bound desensitized state conformation II, which has not been described before. We speculate that the adjusted purification conditions (depletion of ATP using apyrase and addition of Ca^2+^-ions) allowed us to capture both conformations.

There are several known disease-causing mutations in P2X2R, yet potential explanations for their action are not fully understood on a structural level (15). Our high-resolution structures of hP2X2R have provided precise visualization of their location and therefore insights to explain their effects (*SI Appendix*, Fig. S8). For example, we speculate from the structures that V60L on TM1 might interrupt interactions between TM1 and TM2 to render the ion channel constitutively open. D273Y in the lower body, just above the transition to the plasma membrane, may have effects on expression levels of the receptor in the cell membrane. Finally, G353R in TM2, directly next to the pore-forming amino acids, might cause alterations of the gate and influence cation permeation through the pore. These speculations are in line with previous, high-quality predictions (15).

Previous studies have revealed the general architecture of four different P2XR subtypes (hP2X1R, hP2X3R, hP2X4R, and rP2X7R) and described how they fit within the “helical recoil” model of P2XR desensitization (35–40, 51). In contrast, structural knowledge of P2X2R has until now, been limited to the low resolution of 15 Å (71). Our study has verified that hP2X2R shares many of the conserved features within the P2XR family. For example, hP2X2R in the apo closed state has an empty orthosteric pocket and the TMD architecture resembles a teepee, whereas in the desensitized state the orthosteric pocket is occupied by ATP and the TMD architecture resembles a cone (51). This agrees with the mechanism proposed from previous P2XR structures, in which ATP binding induces a series of structural changes that tighten the binding pocket, expands the TM α-helices to open the channel, and subsequently recoils TM2 into a desensitized state with a closed pore (35, 51). Although we were able to determine and visualize the TMs in hP2X2R almost completely, intracellular residues at the cytoplasmic ends of the TMD previously observed in the apo closed state structures of hP2X4R and rP2X7R as well as the ATP-bound open state structures of hP2X3R and rP2X7R (defined as the cytoplasmic cap) were not defined in any of our hP2X2R cryo-EM reconstructions (29, 35, 36, 39, 40). In the case of our determined hP2X2R structures, the lack of a cytoplasmic cap in the apo closed state might be a result of insufficient lipid composition to stabilize the domain, or the cap is transiently formed as proposed for fast desensitizing P2XRs (35, 40). Note that none of the desensitized state structures for any P2XR has contained a cytoplasmic cap (35, 38, 40, 41).

We have also provided insight into some unique structural characteristics of the hP2X2R, as described below. For example, although the orthosteric binding site is highly conserved among P2XRs, coordinating ATP with four lysine residues (for hP2X2R: K81, K83, K200, and K319), a threonine (T196), an asparagine (N300), and an arginine (R302), there are two interactions (L198 and K224) that the hP2X2R only shares with those P2XR subtypes exhibiting slow or nondesensitizing kinetics (hP2X4R and rP2X7R) (Table 2 and *SI Appendix*, Fig. S26). The hydrophobic interaction of a leucine (L198 in hP2X2R) with the adenine core is only observed in the hP2X4R and rP2X7R (L188 and L191, respectively) but not in hP2X1R and hP2X3R where a phenylalanine (F188 and F174, respectively) is found at this position. The weaker hydrophobic interaction with the leucine as compared to phenylalanine might contribute to the weaker apparent affinity of ATP for hP2X2R, hP2X4R, and rP2X7R (5).

**Table 2.** Amino acid residues in the orthosteric binding pockets of various P2XR subtypes whose structures were determined previously^*^


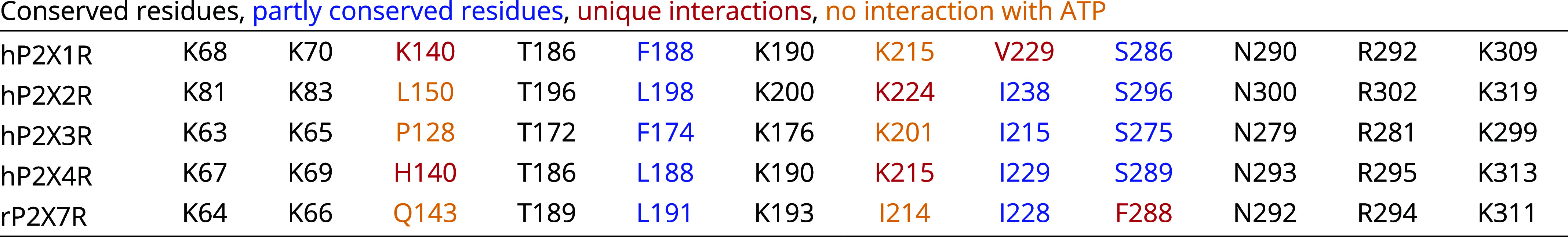

^*^h—human, r—rat. PDB IDs: 9C2B (hP2X1R, ATP-desensitized) (38), 5SVL (hP2X3R, ATP-desensitized) (35), 9C48 (hP2X4R, ATP-desensitized) (40), 6U9W (rP2X7R, ATP-open) (36).

The second unique interaction of the hP2X2R with ATP is solely shared by the hP2X4R. In both orthosteric binding pockets an additional hydrogen bond is formed between a lysine (K224 in hP2X2R and K215 in hP2X4R) and the ribose of ATP. Although lysine residues are also present in hP2X1R and hP2X3R at an equivalent position (K215 and K201 in the hP2X1R and hP2X3R, respectively), the side chain ammonium groups cannot interact with the ribose of ATP because the Cα-backbone of the dorsal fin domain is >1.5 Å farther away from ATP as compared to the structure of the hP2X2R (as calculated by the distance between the 3′-hydroxy-group of the ribose moiety of ATP and the Cα of K224 in hP2X2R, the Cα of K215 in hP2X1R (PDB ID: 9C2B), and the Cα of K201 in hP2X3R (PDB ID: 5SVL))(35, 38).

Finally, in contrast to hP2X1R, hP2X3R, and hP2X4R, no cryo-EM density for a cation was observed in the orthosteric pocket of our hP2X2R reconstructions, suggesting that free anionic ATP^4−^ rather than MgATP^2−^ binds to P2X2R (35, 38, 40, 41, 43, 64). These structural data agree with previously reported as well as current electrophysiological studies demonstrating that hP2X2R has a higher apparent affinity for ATP^4−^ compared to MgATP^2−^, which is a unique feature of the receptor (57, 64). Comparison of the hP2X1R, hP2X3R, and hP2X4R to the hP2X2R on a structural level revealed notable differences between these subtypes in the “Mg^2+^-coordinating region” (*SI Appendix*, Fig. S13). However, the introduction of analogous residues, present in hP2X3R and hP2X4R, into the hP2X2R (G181D and A312T) did not significantly alter the apparent affinities of the hP2X2R for ATP^4−^ or MgATP^2−^. Further studies are required to understand the molecular basis of subtype-selective Mg^2+^-coordination in other P2XR subtypes, and its absence in the hP2X2R (*SI Appendix*, Figs. S13 and S14).

**Fig. 5.**
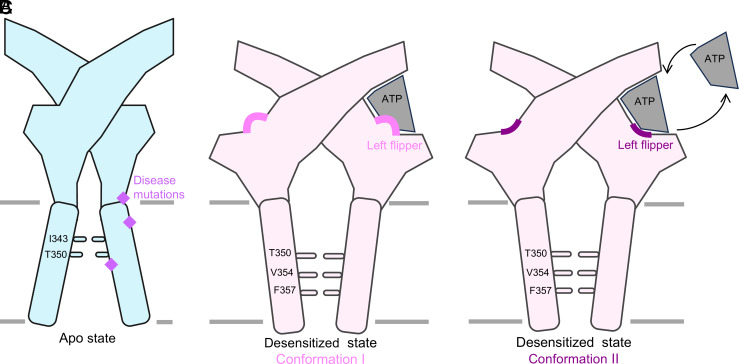
Cartoon models summarizing the findings from the structural studies of the hP2X2R. For simplicity, only two P2X2 protomers and only one ATP molecule are shown. (*A*) Apo state with a closed pore. Residues I343 and T350 form the primary and secondary gate, respectively. Due to the shallow depth of the gate, the overall shape of the transmembrane domain resembles a teepee. (*B* and *C*) Two different desensitized state conformations with ATP bound in the orthosteric binding pocket and a closed pore. Residues T350, V354, and F357 form the gate. Due to the deeper gate, the overall shape of the transmembrane domain in the ATP-bound desensitized state resembles a cone. (*B*) Canonical ATP-bound desensitized state structure, conformation I, with the left flipper domain (colored in pink) interacting with ATP. (*C*) Alternate ATP-bound desensitized state structure, conformation II, in which ATP does not directly interact with the left flipper domain (colored in purple). The structure of conformation II suggests a path by which ATP could enter or leave the orthosteric pocket. The scheme was created with BioRender.com.

It has previously been hypothesized that several conformational changes are required for ATP to enter or leave its binding pocket and activate P2XRs (37, 51). Importantly, our structures of hP2X2R in two different ATP-bound desensitized state conformations suggest that movement of the left flipper might play a role in the association and/or dissociation process of ATP binding (Fig. 5). Indeed, MD simulation studies confirmed that the left flipper in the apo closed state of the receptor is flexible, and ATP-bound desensitized state conformations I and II can interconvert. This is in line with previous mutational studies of the rat P2X2R where the replacement of S284 on the left flipper by alanine (S296A in hP2X2R) was shown to have an effect on the apparent affinity for ATP (13). While our MD simulations do not fully sample an equilibrated exchange cycle of ATP binding and unbinding, the current study nonetheless captures both the formation and loss of the S296–ATP contact (conformation I and II, respectively). These observed transitions across five replicates support the presence of distinct interaction modes. Thus, we speculate that conformation II captures the receptor in a transitionary state where the movement of the loop might facilitate release of ATP from the orthosteric pocket in the desensitized state of hP2X2R.

Taken together, our work contributes to the understanding of the molecular assembly of the P2X2R (Fig. 5). These high-resolution structures of hP2X2R reveal distinct molecular features of this P2XR subtype, providing a basis for rational design of ligands that could potentially treat inflammatory diseases and hearing loss.

## Materials and Methods

### Protein Expression and Membrane Preparation.

The full-length wild-type human P2X2 gene was subcloned into the pEG BacMam vector (72) with a C-terminal human rhinovirus 3C (HRV 3C) protease cleavage site, enhanced GFP (eGFP), and an octahistidine tag. These tags were cleaved off prior to structural determination. All plasmids were verified by sequencing.

A previously published, optimized protocol for baculovirus-mediated gene transduction and protein expression in mammalian cells was used (72). Briefly, the human P2X2 BacMam was transformed into DH10Bac *Escherichia coli* (*E. coli*) (Gibco, USA), isolated using the QIAquick PCR Purification Kit (QIAGEN), and transfected into S*f*9 cells for amplification. The amplified recombinant baculovirus was used to infect HEK cells (HEK293S GnTI^−^). Infected cells (6.4 L) were incubated at 37 °C and 8% CO_2_ for 16 h and then sodium butyrate (10 mM final concentration) was added and cells transferred to 30 °C for an additional 48 h. More details regarding cell culture can be found in the *SI Appendix*.

To achieve the ATP-bound desensitized state structure in conformation I, cells were collected and washed with phosphate-buffered saline (PBS) (137 mM NaCl, 2.7 mM KCl, 8 mM Na_2_HPO_4_, 2 mM KH_2_PO_4_). After resuspension in tris-buffered saline (TBS) (50 mM Tris, 150 mM NaCl) with a pH of 7.0 containing protease inhibitors [1 mM phenylmethylsulfonyl fluoride (PMSF), 0.05 mg/mL aprotinin, 2 mg/mL pepstatin A, and 2 mg/mL of leupeptin], cells were lysed by sonication. For the apo closed and ATP-bound desensitized state structure in conformation II, 0.25 units/mL of apyrase (Sigma-Aldrich) and 5 mM CaCl_2_ were additionally added. After removal of cellular debris by centrifugation, the supernatant was ultracentrifuged to collect the membranes.

### Protein Purification.

The membrane fraction was dounce homogenized in TBS buffer containing 15% glycerol and then solubilized for 1.5 h in 40 mM dodecyl-β-D-maltoside (DDM) and 8 mM cholesteryl hemisuccinate (CHS). The soluble fraction was isolated using ultracentrifugation, loaded onto TALON resin (Takara), pre-equilibrated with washing buffer (TBS plus 5% glycerol, 1 mM DDM, 0.2 mM CHS, pH 8.0) containing 10 mM imidazole, and packed into a XK-16 column. The resin was first washed with 20 mL of buffer containing 20 mM imidazole, followed by 25 mL buffer containing 30 mM imidazole. Proteins were eluted with buffer containing 250 mM imidazole and concentrated. Tris-(2-carboxyethyl)phosphine (TCEP) was added to reach a final concentration of 100 μM, and fusion proteins were digested by HRV 3C protease (1:25, w/w) overnight.

Cleaved proteins were separated using a Superose 6 10/300 GL column pre-equilibrated with 20 mM HEPES, 100 mM NaCl, 0.5 mM DDM, and 0.1 mM CHS at pH 7.0 at a flow rate of 0.5 mL/min. After the fractions eluted, TCEP (final concentration of 100 µM) was added and peak fractions were collected and concentrated to ~5 mg/mL. The protein samples were verified by SDS-PAGE analysis and fluorescence size-exclusion chromatography.

### Cryoelectron Microscopy Sample Preparation, Data Acquisition, and Data Processing.

To prepare the cryo-EM grids, the protein sample (2.5 μL) was applied to glow-discharged (15 mA, 1 min) Quantifoil holey carbon grids (Cu 1.2/1.3 300 mesh), which were blotted for 1.5 s or 2 s under 100% humidity at 4 °C. The grids were then plunge-frozen in liquid ethane, cooled by liquid nitrogen using an FEI Vitrobot Mark IV, and stored under liquid nitrogen until data acquisition. No ATP was added to the protein.

Cryo-EM data for each conformational state were collected on Titan Krios microscopes (FEI) operated at 300 kV at the Pacific Northwest Center for Cryo-EM (PNCC). Datasets were all acquired using an energy filter (slit width of 10 eV for the apo closed and the ATP-bound desensitized conformation II or 20 eV for the ATP-bound desensitized conformation I) and a Gatan K3 direct-electron detector. All movies were collected in superresolution mode at a nominal magnification of 130,000×, corresponding to a physical pixel size of ~0.648 Å/pixel, using a defocus range of –0.9 to –1.5 µm, 50 frames, and total dose of ~45 e^−^/ Å^2^. Each dataset utilized “multi-shot” and “multi-hole” collection schemes driven by serialEM (73).

Data processing was conducted as described in *SI Appendix*, Fig. S1 using cryoSPARC (74). Briefly, movies were motion corrected, CTF parameters estimated, micrographs curated, particles template picked, inspected, and then extracted and binned to a pixel size of ~1.3 Å/pixel. Particles were sent directly to iterative 3D classification (skipping 2D classification) using ab-initio jobs followed by heterogenous classifications. After final particle stacks were obtained, further CTF corrections and nonuniform refinements were performed at the physical pixel size to generate the final reconstructions. Map sharpening was performed using DeepEMhancer (75).

### Model Building.

The initial homology models of the hP2X2R in the apo closed and ATP-bound desensitized states were generated with SWISS-MODEL using the apo closed state of zfP2X4R or the ATP-bound desensitized state of hP2X3R [PDB IDs: 4DW0 or 5SVL, respectively (35, 42, 76)]. Models were subsequently built using Coot and real-space refinements were performed within PHENIX (77, 78). Limited glycosylations were included in the models when justified by cryo-EM density. In each model, residues or their side chains are not included if they were missing from the cryo-EM density. Model quality was evaluated by MolProbity (*SI Appendix*, Table S1) (79). All figures of structures were prepared using UCSF ChimeraX (47, 48).

### Two-Electrode Voltage Clamping.

The pcDNA 3.1× plasmid encoding for wild-type hP2X2R used for electrophysiology was a full-length wild-type hP2X2R with no GFP, protease sites, or affinity tags (equivalent to the receptor used for structure determination) (80). Mutations to the wild-type hP2X2R construct were generated using QuikChange XL mutagenesis kits and included: G181D and G181D/A182T. RNA was synthesized using the mMESSAGE mMACHINE® T7 Ultra Kit (Thermo Fisher Scientific). Oocytes, purchased from Ecocyte Bioscience, were injected with 50 nL of 50 ng/µL mRNA. Until recording (~20 h of expression time), oocytes were stored at 18 °C in oocyte storage buffer (88 mM NaCl, 1 mM KCl, 2.4 mM NaHCO_3_, 10 mM HEPES-NaOH pH 7.4, 0.33 mM Ca(NO_3_)_2_ · 4 H_2_O, 0.41 mM CaCl_2_ · 2 H_2_O, MgSO_4_ · 7 H_2_O, 250 mg/L amikacin, and 150 mg/L gentamycin).

For concentration–response curves, calcium-free recording buffers at pH value 7.4 with Mg^2+^ (100 mM NaCl, 2.5 mM KCl, 5 mM MgCl_2_, 5 mM HEPES, and 0.1 mM flufenamic acid) or without Mg^2+^ (100 mM NaCl, 2.5 mM KCl, 0.1 mM ethylenediaminetetraacetic acid (EDTA), 5 mM HEPES, and 0.1 mM flufenamic acid) were used. Recording electrode pipettes (1 to 2.5 MΩ) were filled with 3 M KCl. Oocytes were voltage-clamped at −60 to −65 mV. The agonist (ATP, Sigma) was dissolved in recording buffer with or without Mg^2+^-ions and diluted in a serial dilution starting at either 1,920 μM or 100 μM, respectively. The ATP-containing buffer was applied for ~10 s at a flow rate of ~5 mL/min. For each recording, the highest concentration of ATP was applied as a control to induce maximal receptor activation, and subsequent recordings at the lower ATP concentrations were normalized as percentage of the maximal current (set as 100%). The oocytes were perfused with buffer for at least 20 s (generally for 2 min) between applications of ATP. Each concentration was determined in at least three biological replicates. To study the desensitization profiles, the recording buffers described above were tested in addition to buffer containing Ca^2+^ (100 mM NaCl, 2.5 mM KCl, 5 mM CaCl_2_, 5 mM HEPES, and 0.1 mM flufenamic acid). Data acquisition was performed using a Warner OC-725C amplifier (Warner Instruments) and pClamp 8.2 software (Molecular Devices) and then exported to Prism 10 (GraphPad).

### MD simulation studies.

The cryo-EM structures of the hP2X2R in the apo closed state and both ATP-bound desensitized states (conformation I and II) were used as starting points for MD simulations. The Maestro suite 2024-1 (Schrödinger Release 2024-1: Schrödinger, LLC, New York, NY, 2024) was used for MD studies, which requires each structure to pass through a fully automated preprocessing protocol. Finally, the resulting structures were autoenergy minimized using OPLS4 force field (81) at a physiological pH value of 7.2 using Epik (82, 83).

Long-range MD simulations were conducted for the three distinct conformations, as described previously (84, 85) using the Desmond MD engine (86), equipped with the OPLS4 force field (87, 88), employing a 2 fs time step integration. Initially, the preprocessed hP2X2R–ATP complex structures were prepared using the system builder panel in Maestro. The systems were set-up by centering the protein within a cubic box, ensuring a minimum distance of 10 Å between the protein surface and the box boundaries. The transmembrane helices were appropriately oriented within a membrane bilayer composed of POPC (1-palmitoyl-2-oleoyl-sn-glycero-3-phosphocholine). The simulation box was then solvated with the TIP3P water model (89), and the system’s net charge was neutralized with Na^+^ and Cl^−^ counterions, maintaining a salt concentration of 0.15 M and a temperature of 300 K. The simulations underwent multistage equilibration as follows: i) Brownian Dynamics in the NVT ensemble at 10 K with small time-steps and heavy-atom restraints on the solute for 100 ps; ii) NVT ensemble at 10 K with small timesteps and heavy-atom restraints for 12 ps; iii) NPT ensemble at 10 K with heavy-atom restraints for 12 ps; iv) further equilibration at 300 K with heavy-atom restraints for 12 ps; and v) final equilibration at 300 K with all restraints removed for 24 ps. Following equilibration, 1 μs production runs were performed for each of the three systems, with five replicates per system.

More details regarding the MD methods (initial structure preparation, trajectory analysis, data visualization, and binding free-energy calculation) can be found in *SI Appendix*.

## Supplementary Material

Appendix 01 (PDF)

Movie S1. Visualization of one representative MD simulation for the hP2X2R in the apo closed state highlighting the dramatic conformational flexibility of the left flipper in the absence of ATP. The movie shows the movements between 540 ns and 800 ns of the representative trajectory. The backbone of the left flipper (residues 288–299) is depicted in orange, with the residues S296, P294, and P290 shown in stick representation. The heteroatoms are shown in color: nitrogen in blue and oxygen in red. Hydrogen atoms are shown in white. Neighboring protein domains are represented by contours in black.

Movie S2. Visualization of one representative MD simulation for the hP2X2R in the canonical ATP-bound desensitized state (conformation I) highlighting the movements of residue S296 and ATP. The movie shows the movements between 600 ns and 800 ns of one representative trajectory. The backbone of the left flipper (residues 288–299) is depicted in blue, with the residues S296, P294, and P290 shown in stick representation. The heteroatoms are shown in color: nitrogen in blue, oxygen in red, and phosphorus in magenta. Hydrogen atoms are shown in white and carbon atoms of ATP in tan. Neighboring protein domains are represented by contours in black. Distances between S296 and the α-phosphate of ATP are labeled in green.

Movie S3. Visualization of one representative MD simulation for the hP2X2R in the alternate ATP-bound desensitized state (conformation II) highlighting the movements of residue S296 and ATP. The movie shows the movements between 600 ns and 750 ns of one representative trajectory. The backbone of the left flipper (residues 288–299) is depicted in dark green, with the residues S296, P294, and P290 shown in stick representation. The heteroatoms are shown in color: nitrogen in blue, oxygen in red, and phosphorus in magenta. Hydrogen atoms are shown in white and carbon atoms of ATP in tan. Neighboring protein domains are represented by contours in black. Distances between S296 and the α-phosphate of ATP are labeled in green.

## Data Availability

All cryo-EM density maps for the full-length wild-type hP2X2R in the apo closed and two ATP-bound desensitized state conformations have been deposited in the Electron Microscopy Data Bank (EMDB) under the accession codes: EMD-46781 (apo closed state) (90), EMD-46782 (canonical ATP-bound desensitized state conformation I) (91), and EMD-46783 (alternate ATP-bound desensitized state conformation II) (92). The corresponding coordinates for the structures have been deposited in the Protein Data Bank under the PDB accession codes 9DDV (apo closed state) (93), 9DDW (canonical ATP-bound desensitized state conformation I) (94), and 9DDX (alternate ATP-bound desensitized state conformation II) (95).
