# Supplementary material for: Subtype-specific structural features of the hearing loss–associated human P2X2 receptor
Source: Proc Natl Acad Sci U S A. 2025 Sep 12;122(37):e2417753122. doi: 10.1073/pnas.2417753122 (PMC12452952; doi:10.1073/pnas.2417753122)
Supplement: Supplementary file 1 — Appendix 01 (PDF) [file pnas.2417753122.sapp.pdf]

PNAS

Supporting Information for

Subtype-specific structural features of the hearing loss-associated human P2X2 receptor

Franka G. Westermann[a,b,c], Adam C. Oken[c], Philip K. E. Granith[d], Parthiban Marimuthu[d], Christa E. Müller[a,b], Steven E. Mansoor[c,e]

a. PharmaCenter Bonn and Pharmaceutical Institute, Department of Pharmaceutical & Medicinal Chemistry, University of Bonn, 53121 Bonn, Germany.
b. Research Training Group 2873, University of Bonn, 53121 Bonn, Germany
c. Department of Chemical Physiology & Biochemistry, Oregon Health & Science University, Portland, Oregon 97239, USA.
d. Pharmaceutical science laboratory (PSL – Pharmacy) and Structural Bioinformatics Laboratory (SBL – Biochemistry), Faculty of Science and Engineering (FNT), Åbo Akademi University, FI-20520 Turku, Finland.
e. Division of Cardiovascular Medicine, Knight Cardiovascular Institute, Oregon Health & Science University, Portland, Oregon 97239, USA.

Corresponding authors:

Steven E. Mansoor
3181 S.W. Sam Jackson Park Road, Portland, OR 97239
+1 503-346-3104
mansoors@ohsu.edu

Christa E. Müller
An der Immenburg 4, 53121 Bonn
+49 228 73-2301
christa.mueller@uni-bonn.de

This PDF file includes:

- Supporting text
- Figures S1 to S26
- Table S1
- Legends for Movies S1 to S3
- SI References

Other supporting materials for this manuscript include the following:

- Movies S1 to S3

Supporting Information Text

Extended methods for cell culture

Spodoptera frugiperda insect cells (*Sf*9) (ThermoFisher Scientific) were cultured and routinely passaged in suspension at 27°C in Sf900 III SFM medium (ThermoFisher Scientific).
HEK293S GnTI^{-} cells were cultured and routinely passaged in FreeStyle 293 Expression Medium (Gibco) at 37°C supplemented with 2% (v/v) fetal bovine serum.

Extended methods for molecular dynamics (MD) simulations

Initial structure preparation

To ensure sufficient embedding of the human P2X2 receptor (hP2X2R) in the membrane bilayer, the experimentally determined structures were extended (now spanning N38 to T365) using MODELLER v10.6 (1). ClustalOmega (https://www.ebi.ac.uk/jdispatcher/msa/clustalo) was used to perform preliminary sequence alignment between the template (cryo-EM structure) and the target sequence (Uniprot: Q9UBL9), while Jalview (2) was used to refine the resulting alignment. MODELLER generated 100 homotrimeric hP2X2R models, while retaining the position of ATP. Structural validation via SAVES v6.0 (https://saves.mbi.ucla.edu/) included Ramachandran plots (Procheck), Cα superposition (Maestro GUI, Schrödinger Inc., NY, USA), ERRAT, and DOPE (Distance Optimized Potential Energies) scores. Models passing stereochemical assessment and closely resembling the cryo-EM structure were selected for MD simulations.

Trajectory analysis

Initially, all trajectories were carefully processed using the *Simulation Quality Analysis* and *Simulation Interaction Diagram* modules embedded in Maestro. Subsequently, to monitor the movement of the left flipper loop located proximal to the orthosteric binding pocket with respect to ATP during the MD simulation, we employed the *interaction count* module available in the Maestro GUI. The atoms involved in the interaction were manually selected in the Maestro GUI which included the hydrogen on the hydroxyl side chain of S296 in the left flipper and an oxygen atom on the α-phosphate group of ATP, which is bound to the orthosteric binding pocket of the hP2X2R.

MD simulation data visualization

All structural visualizations, investigations, and rendering of interaction figures were performed using the Maestro GUI panel. All results from the trajectories were plotted using Python 3.7.

Molecular Mechanics with Generalized Born Surface Area and free binding energy calculation

To calculate the binding free energies of the protein-ligand complex and estimate per-residue decomposition (PRD), the Molecular Mechanics with Generalized Born Surface Area (MM/GBSA) method was employed. To achieve this, the *thermal_mmgbsa.py* script available in Maestro (3) was applied to the entire 1 µs trajectory with an interval of 4 frames for a total of 625 frames. The energy contributed by each residue present in the left flipper loop over the simulation was visualized using heatmaps.

The MM/GBSA ΔG_{bind} energy values were calculated by the following equation: MM/GBSA $\Delta G_{bind} = E_{Complex} - E_{Ligand} - E_{Receptor}$. The energy estimation based on the MM/GBSA method in Maestro lacks the conformational entropic value, which is not computed.

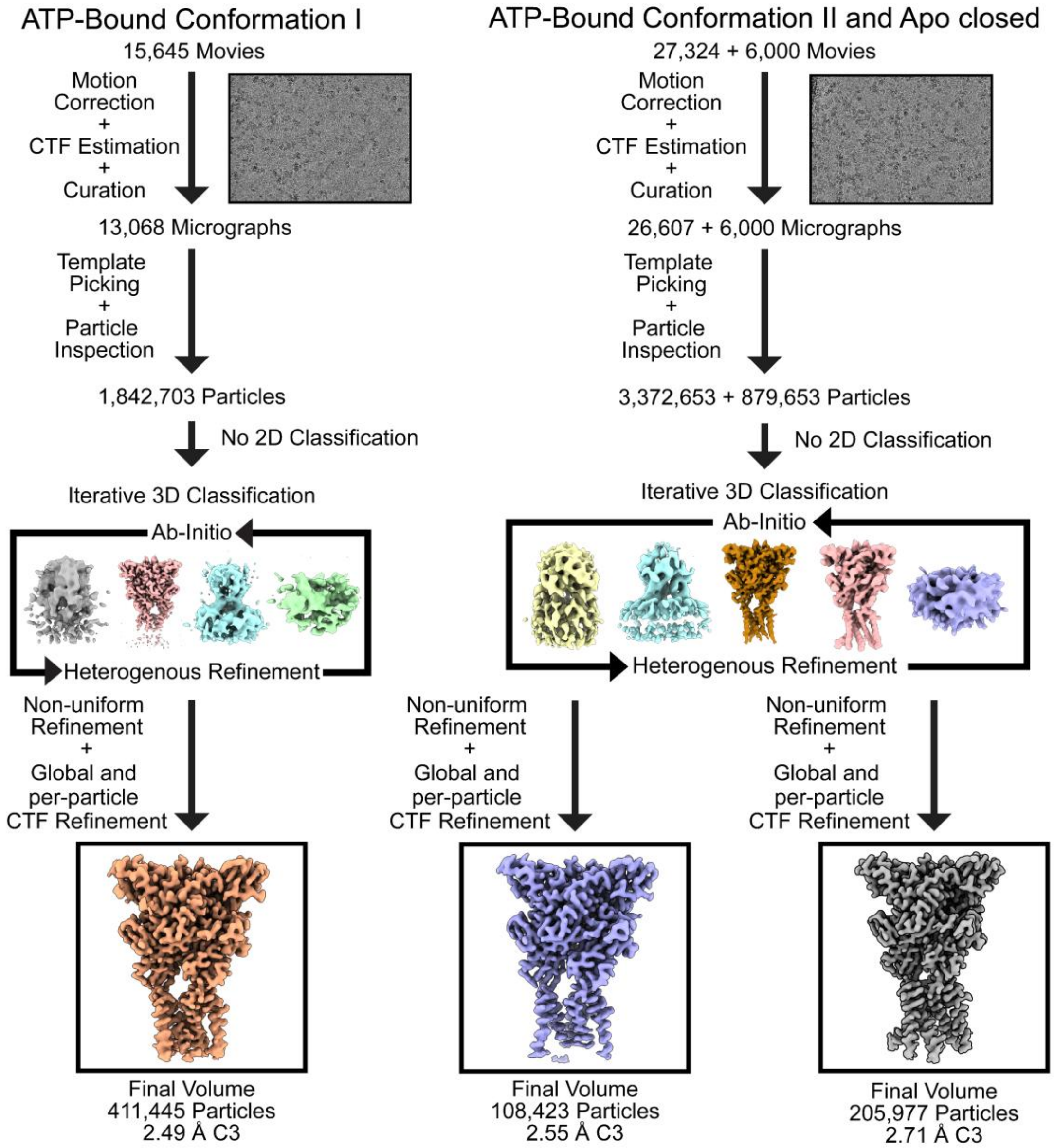

Fig. S1. Cryo-EM processing pipeline for hP2X2R reconstructions. Cryo-EM image processing strategies for the canonical ATP-bound desensitized state (conformation I), the alternate ATP-bound desensitized state (conformation II), and the apo closed state using cryoSPARC (4). After data acquisition, movies were motion corrected and CTF parameters estimated. Micrographs were then manually curated and template picked using 2D classes generated from an initial 3D volume. Particles were inspected, extracted, and sent directly to iterative 3D classification (skipping 2D classification) using ab-initio jobs to generate initial reconstructions that composed the inputs of heterogenous classifications. After final particle stacks were obtained, further CTF corrections and non-uniform refinements were performed at the physical pixel size to generate the final reconstructions. The final set of particles resulted in cryo-EM reconstructions at 2.49 Å, 2.55 Å and 2.71 Å resolution for the canonical ATP-bound desensitized state (conformation I), the alternate ATP-bound desensitized state (conformation II), and the apo closed state structures, respectively.

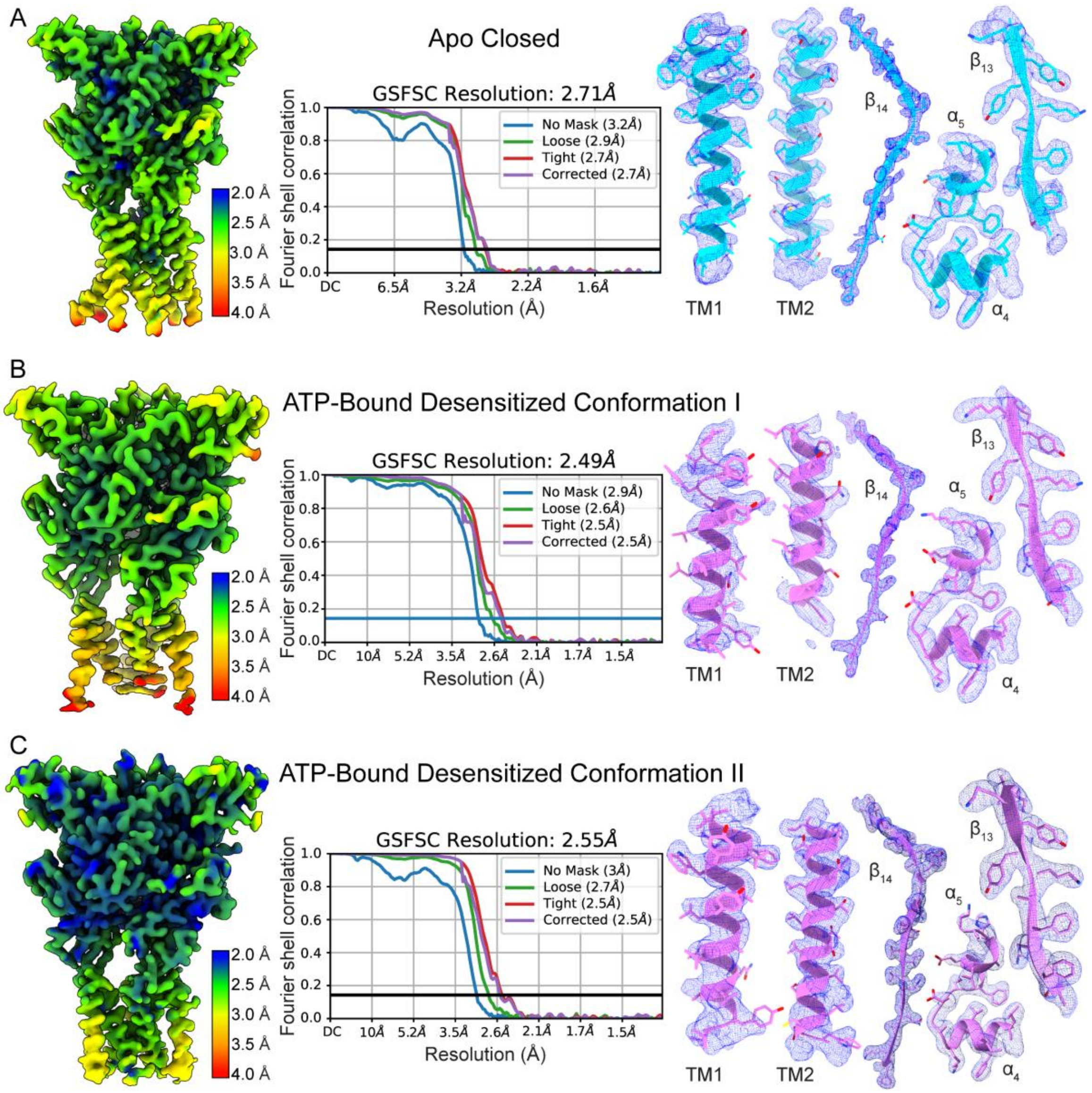

Fig. S2. Local resolution, Fourier shell correlation (FSC) plots, and map-to-model fit for the cryo-EM maps of the hP2X2R in the apo closed and both ATP-bound desensitized states. (*A-C left*) Local resolution estimates of the reconstructions shown in side view, parallel to the membrane, for the (*A*) apo closed state, (*B*) canonical ATP-bound desensitized state (conformation I), and (*C*) an alternate ATP-bound desensitized state (conformation II). All local resolution plots range from 2.0 Å (blue) to 4.0 Å (red). (*A-C middle*) Gold-standard Fourier shell correlation (FSC) curves for the electron microscopy maps of the apo closed state (*A*), canonical ATP-bound desensitized state (conformation I) (*B*), and an alternate ATP-bound desensitized state (conformation II) (*C*). The resolutions stated are at an FSC = 0.143. (*A-C right*) Selected elements of the hP2X2R in the apo closed state (*A*, blue mesh and blue ribbon), conformation I of the ATP-bound desensitized state (*B*, blue mesh and purple ribbon), and conformation II of the ATP-bound desensitized state (*C*, blue mesh and purple ribbon) highlighting good map-to-model fits.

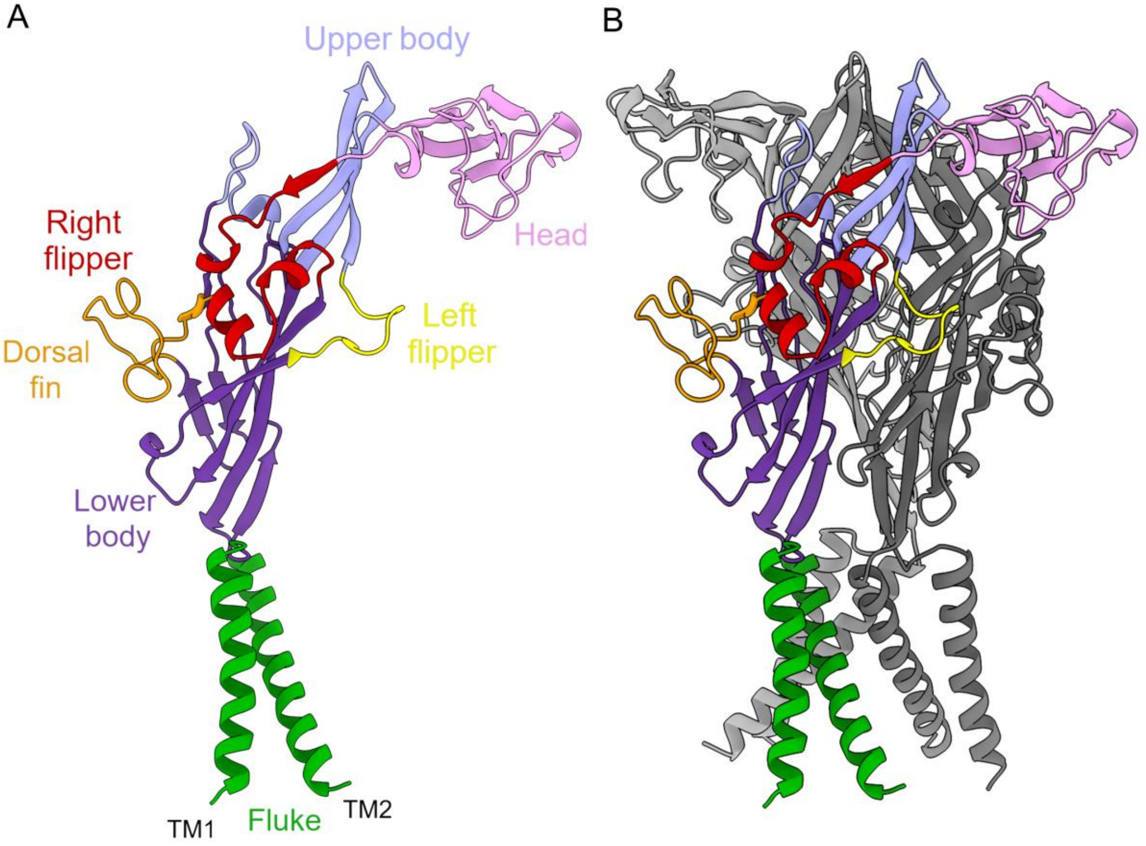

Fig. S3. Purinergic receptor domain names. (*A* and *B*) Ribbon representation of a single protomer (*A*) and a full trimer (*B*) of hP2X2R shown in the apo closed state, colored by domain. Each protomer is imagined to resemble a breaching dolphin, and the domains are named according to dolphin anatomy (5). The second and third protomers in panel *B* of the trimeric receptor are colored in light gray and dark gray, respectively.

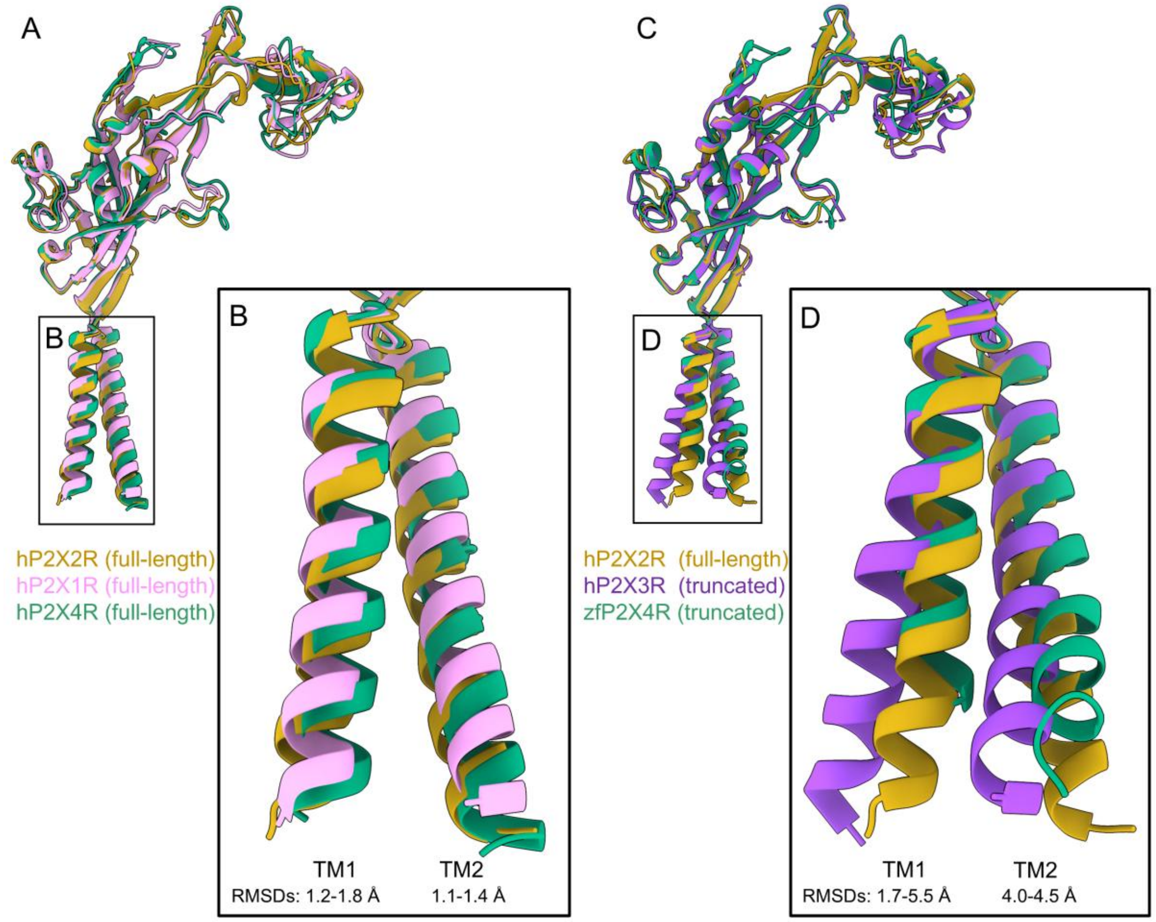

Fig. S4. Comparison of apo closed state structures for full-length hP2X2R to published structures of other P2XR orthologs derived from both full-length and truncated constructs. (*A*) Ribbon representation of one hP2X2R protomer (gold) aligned with one protomer of hP2X1R (pink, PDB ID: 9C2A (6)) and one protomer of hP2X4R (green, PDB ID: 9BQH (7)). (*B*) Magnified view of the aligned transmembrane domains of panel *A* which exhibit RMSDs of 1.1-1.8 Å. (*C*) Ribbon representation of one hP2X2R protomer (gold) aligned with one protomer of hP2X3R (purple, PDB ID: 5SVJ (8)) and one protomer of zfP2X4R (green, PDB ID: 4DW0 (9)). (*D*) Magnified view of the overlayed transmembrane domains of panel *C* which exhibit RMSDs of 1.7-5.5 Å as calculated with ChimeraX (10, 11).

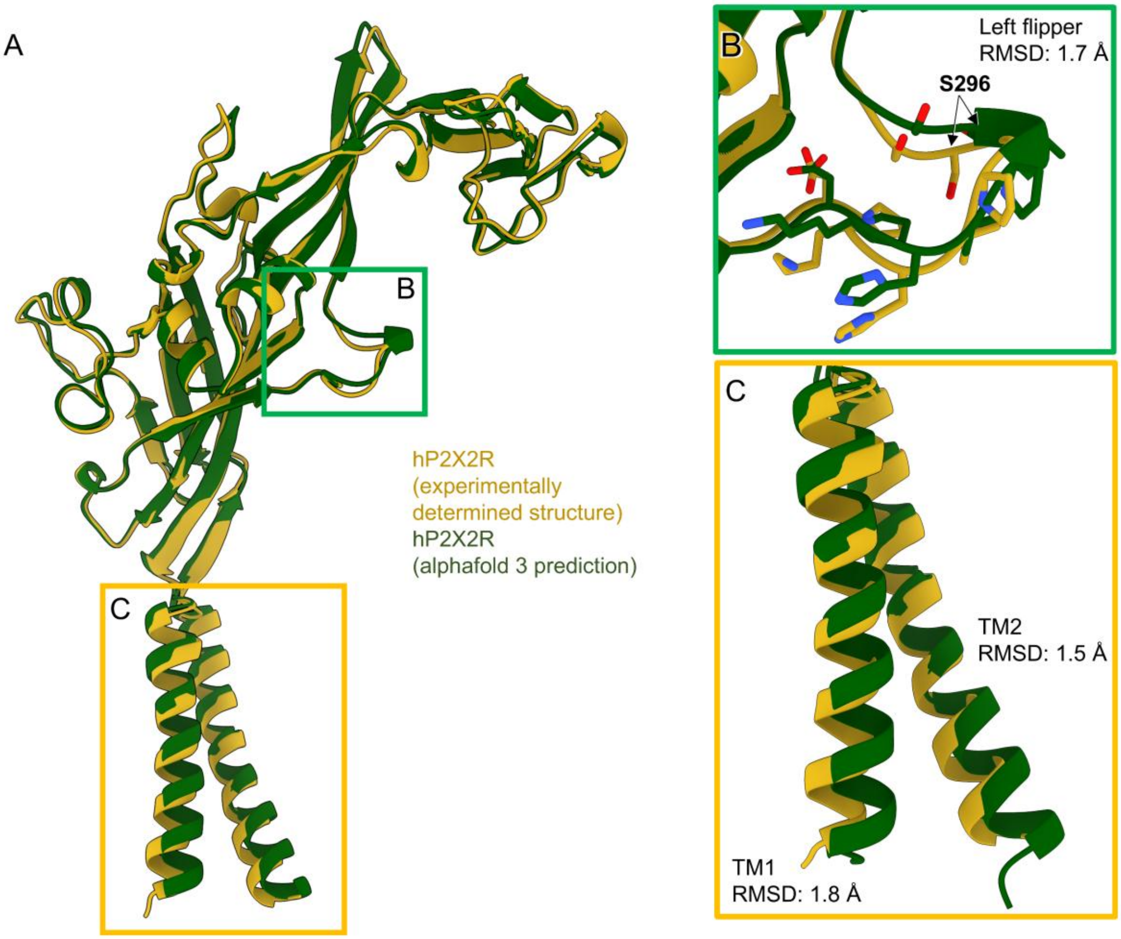

Fig. S5. Comparison between the AlphaFold 3 prediction and the cryo-EM structure of a hP2X2 protomer in the apo closed state conformation. (*A*) Ribbon representation of one hP2X2 protomer from the AlphaFold 3 prediction (12) in green overlayed with one protomer from our cryo-EM structure in gold. The green and yellow boxes highlight two areas with notable deviations between the two models. The RMSDs were calculated with ChimeraX (10, 11). (*B*) Magnified view of the green box in panel *A* highlighting the differences in the left flipper between experimental cryo-EM and predicted AlphaFold 3 structures. The left flipper has an RMSD of 1.7 Å between the AlphaFold 3 prediction and the cryo-EM structure. The side chains shown are colored by atom: nitrogen in blue and oxygen in red. (C) Magnified view of the yellow box in panel *A* highlighting the differences in the transmembrane domain between experimental cryo-EM and predicted AlphaFold 3 structures. TM1 has an RMSD of 1.8 Å between the AlphaFold 3 prediction and the cryo-EM structures while TM2 has an RMSD of 1.5 Å.

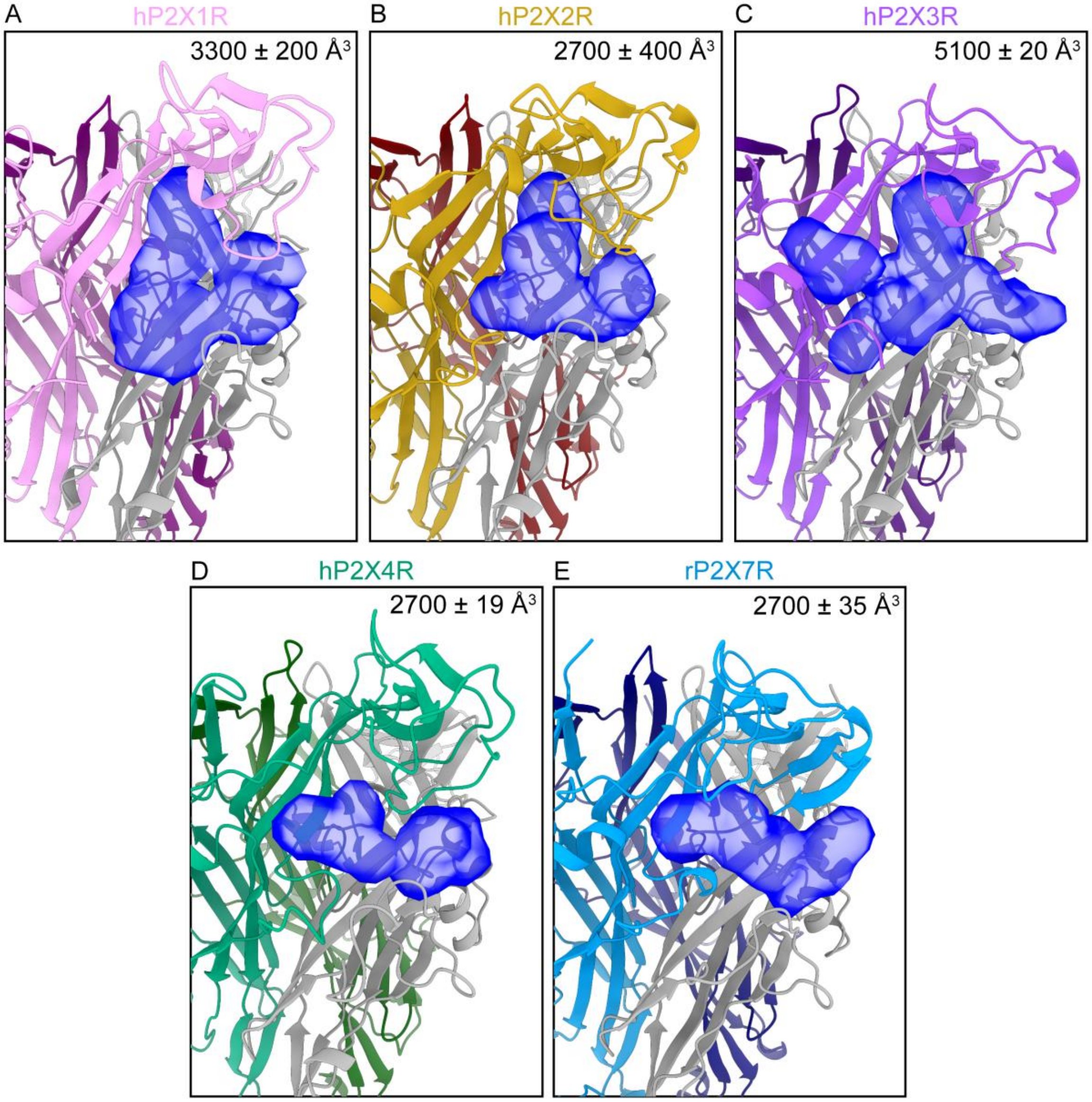

Fig. S6. Comparison of the unoccupied orthosteric ATP-binding sites between P2XR subtypes in the apo closed state conformation. (*A-E*) Ribbon representation of P2XR subtypes in the apo closed state comparing size differences of the empty orthosteric pocket. The surface accessible size of the orthosteric pockets were calculated using Fpocket (13) and are represented as blue volumes. Each protomer is colored differently: (*A*) hP2X1R (shades of pink, PDB ID: 9C2A (6)), (*B*) hP2X2R (shades of gold), (*C*) hP2X3R (shades of purple, PDB ID: 5SVJ (8)), (*D*) hP2X4R (shades of green, PDB ID: 9BQH (7)), and (*E*) rP2X7R (shades of blue, PDB ID: 8TR5 (14)).

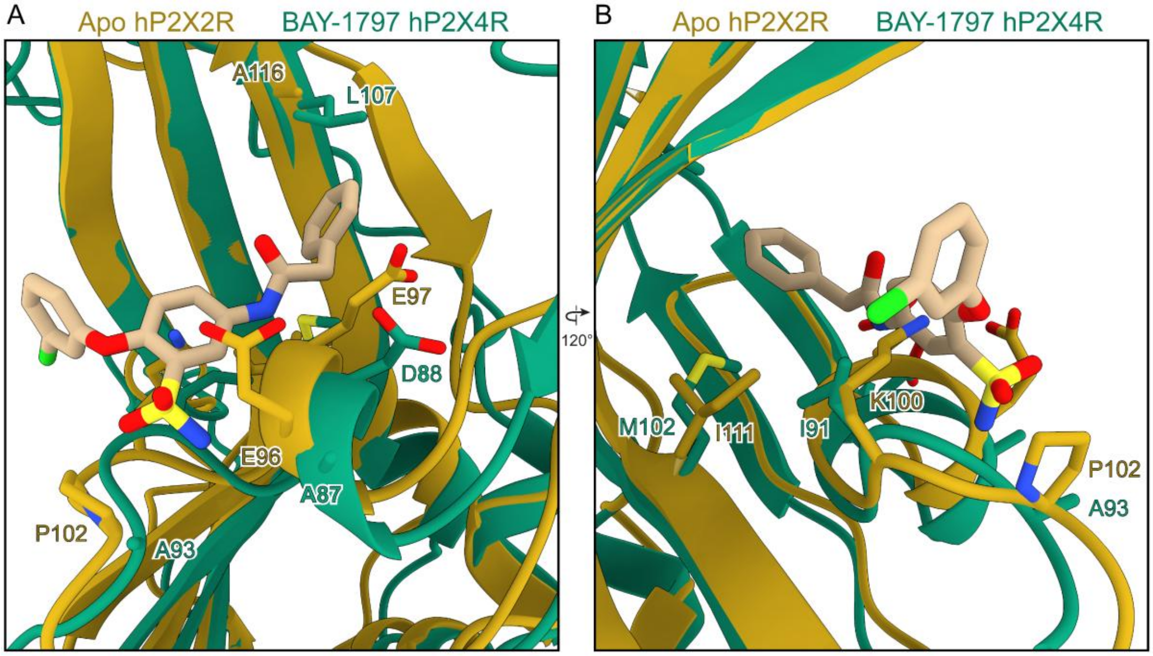

Fig. S7. Comparison between the BAY-1797-bound allosteric pocket of the hP2X4R and the analogous location in the apo closed state structure of hP2X2R. (*A* and *B*) Magnified view of the BAY-1797-bound allosteric pocket within the hP2X4R (PDB ID: 9BQI (7)) overlayed with the apo closed state structure of the hP2X2R. Residues within the allosteric pocket of the hP2X4R are shown in green and residues in the analogous positions within the hP2X2R are shown in gold. *(A)* View highlighting the different residues in hP2X2R compared to hP2X4R. Residues A87, D88, A93, and L107 in hP2X4R structurally correlate to the larger side chains of E96, E97, P102, and A116 in the hP2X2R, respectively. *(B)* Rotated (120° counter-clockwise around the y-axis) view of panel *A* highlighting additional residue differences including I91 and M102 in hP2X4R which structurally correlate to K100 and I111 in the hP2X2R, respectively. A clash between the side chain of residue K100 in hP2X2R and an allosteric ligand bound in the same site as in hP2X4R is predicted. This likely contributes to the fact that BAY-1797 shows weak inhibition at other P2XR subtypes (15). The side chains of hP2X2R and hP2X4R shown and the heteroatoms of the BAY-1797 molecule are colored by atom: nitrogen in blue, oxygen in red, chlorine in green, and sulfur in yellow. The carbon atoms of BAY-1797 are shown in tan.

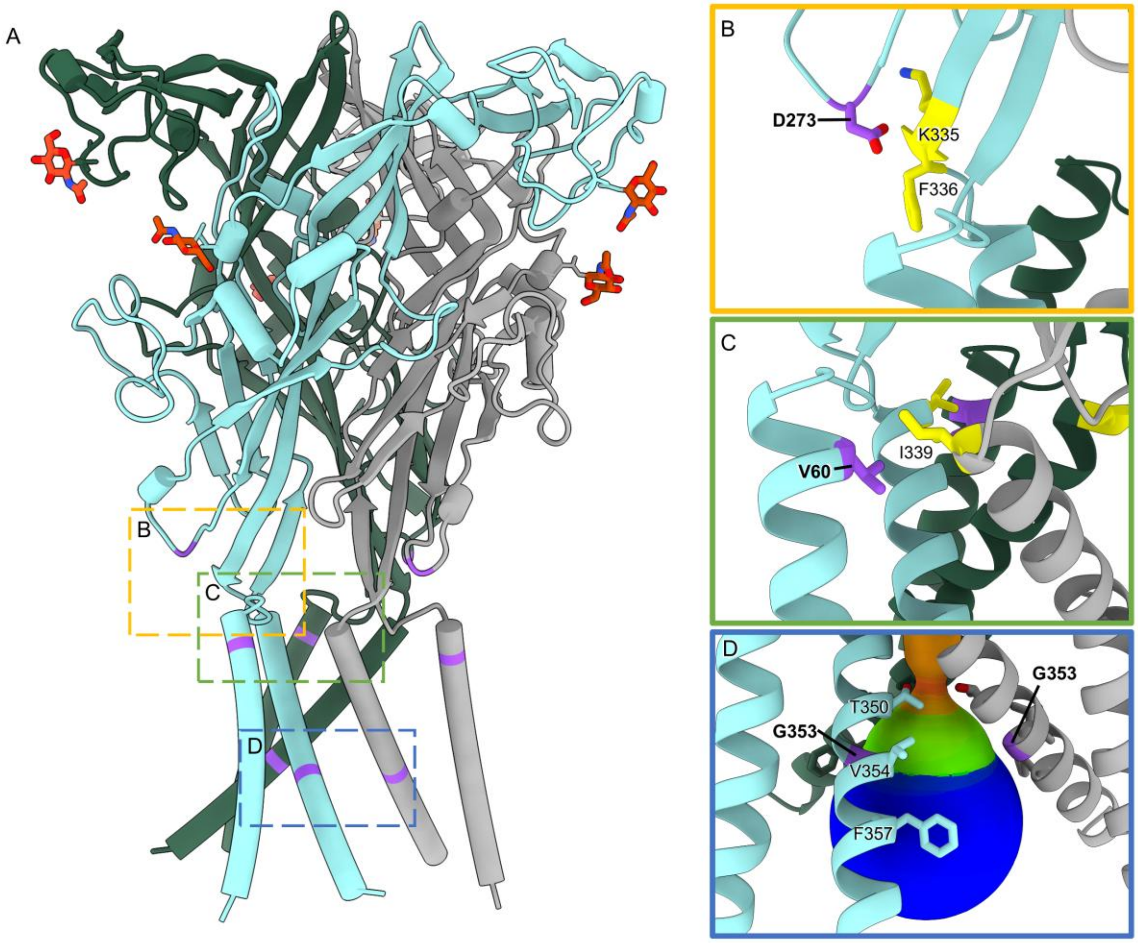

Fig. S8. Locations of the residues in the hP2X2R that are reported to cause hearing loss when mutated and their neighboring interaction partners. The hearing loss-associated mutations are V60L, D273Y, and G353R (16). (*A*) Apo closed state structure of the hP2X2R with the locations of residues V60, D273, and G353 indicated in purple. Glycosylation is colored by atom: carbon in orange, nitrogen in blue, and oxygen in red. (*B-D*) Close-up view of the residues (*B*) D273, (*C*) V60, (*D*) G353 indicated in purple with their neighboring interaction partners in yellow. Functional groups of side chains shown are colored by atom: nitrogen in blue and oxygen in red.

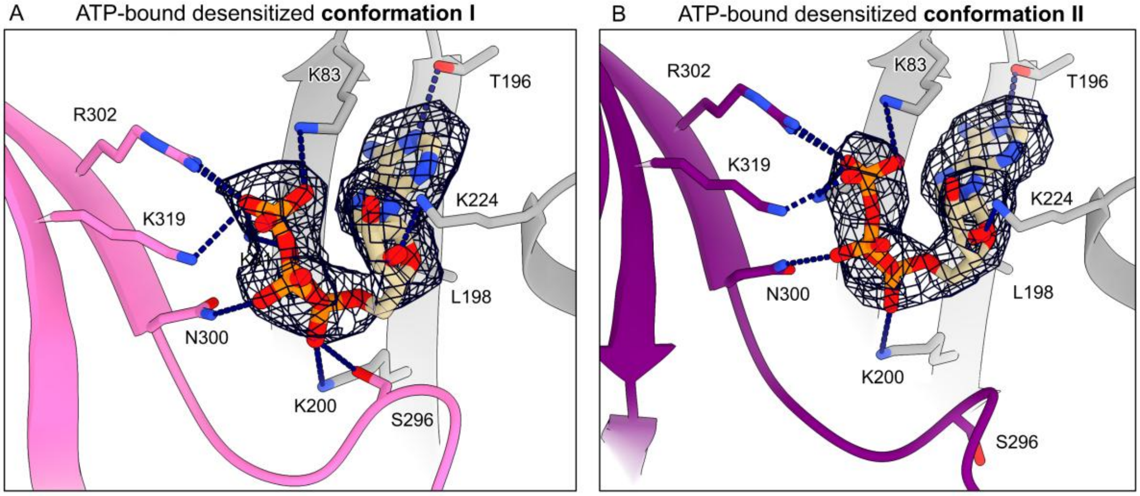

Fig. S9. Strong cryo-EM density around ATP in the orthosteric pocket for both ATP-bound desensitized state conformations. (*A*) Magnified view of Fig. 2*B* highlighting the cryo-EM density for ATP bound within the orthosteric pocket in the canonical ATP-bound desensitized state structure (conformation I) of hP2X2R. (*B*) Same view as panel *A* highlighting the cryo-EM density for ATP bound within the orthosteric pocket in the alternate ATP-bound desensitized state structure (conformation II) of hP2X2R. The cryo-EM density is shown in black mesh. The side chains shown and the heteroatoms of ATP are colored by atom: nitrogen in blue, oxygen in red, and phosphorus in orange. The carbon atoms of ATP are shown in tan.

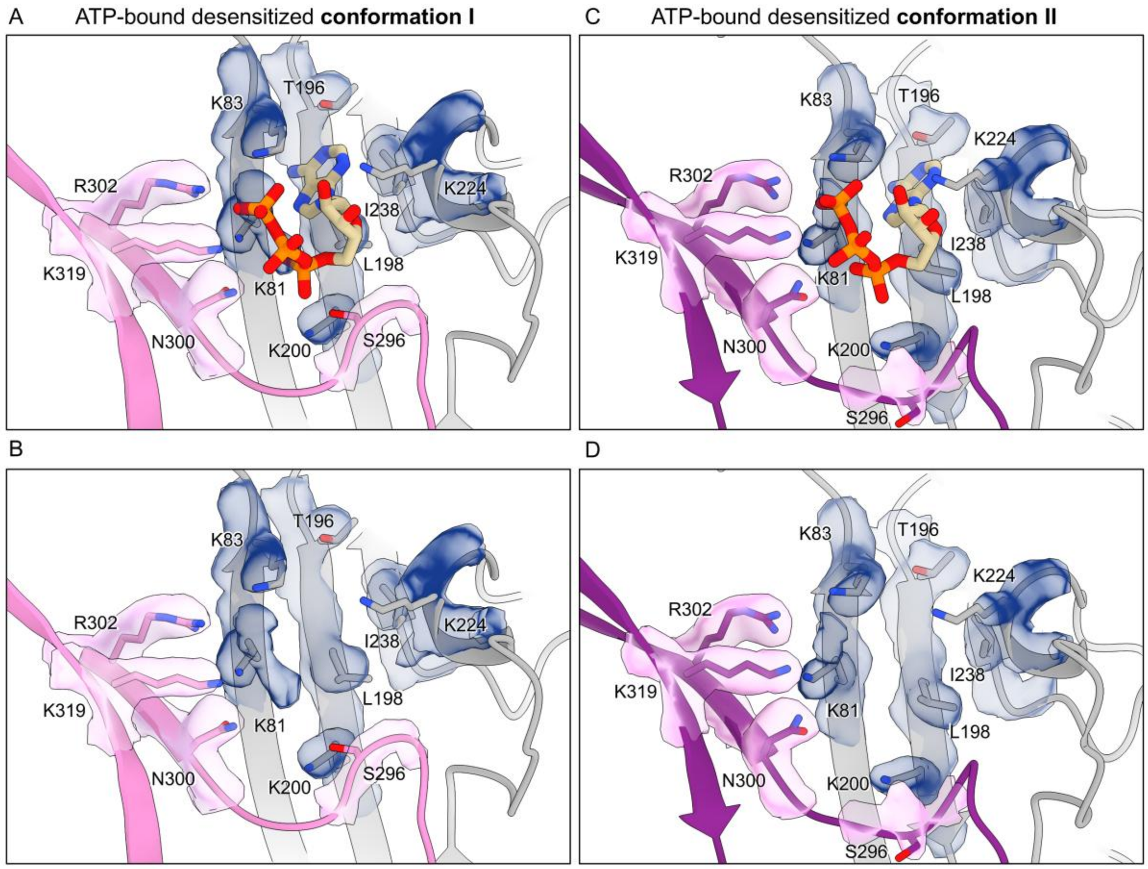

Fig. S10. Side chain cryo-EM density of residues that interact with ATP within the orthosteric binding pocket of the hP2X2R in both ATP-bound desensitized state conformations. (*A* and *B*) Magnified view of Fig. 2*B* showing the cryo-EM density for the side chains that interact with ATP in the canonical ATP-bound desensitized state structure (conformation I) of hP2X2R (*A*) with ATP shown, (*B*) with ATP hidden for visual clarity. (*C* and *D*) Magnified view of Fig. 2*B* showing the cryo-EM density for the side chains that interact with ATP in the alternate ATP-bound desensitized state structure (conformation II) of hP2X2R (*C*) with ATP shown, (*D*) with ATP hidden for visual clarity. The cryo-EM density for the side chains is shown as a transparent surface. The side chains shown in the model and the heteroatoms of ATP are colored by atom: nitrogen in blue, oxygen in red, and phosphorus in orange. The carbon atoms of ATP are shown in tan.

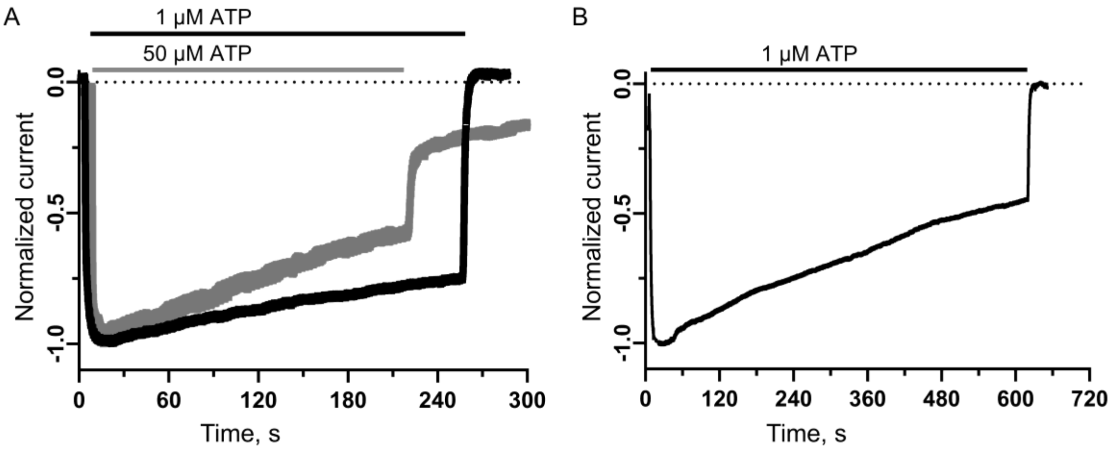

Fig. S11. Representative desensitization profiles of the hP2X2R expressed in *Xenopus* oocytes and measured by TEVC in the absence of divalent cations. (*A*) Consistent with previous electrophysiology studies, the hP2X2R exhibits slow desensitization following activation by ATP. The desensitization is faster when activated by 50 µM ATP (gray trace) compared to 1 µM ATP (black trace). (*B*) Desensitization observed following extended application of 1 µM ATP. Even at a concentration of 1 µM of ATP, the hP2X2R shows > 50% desensitization after 600 s (10 min) of agonist application.

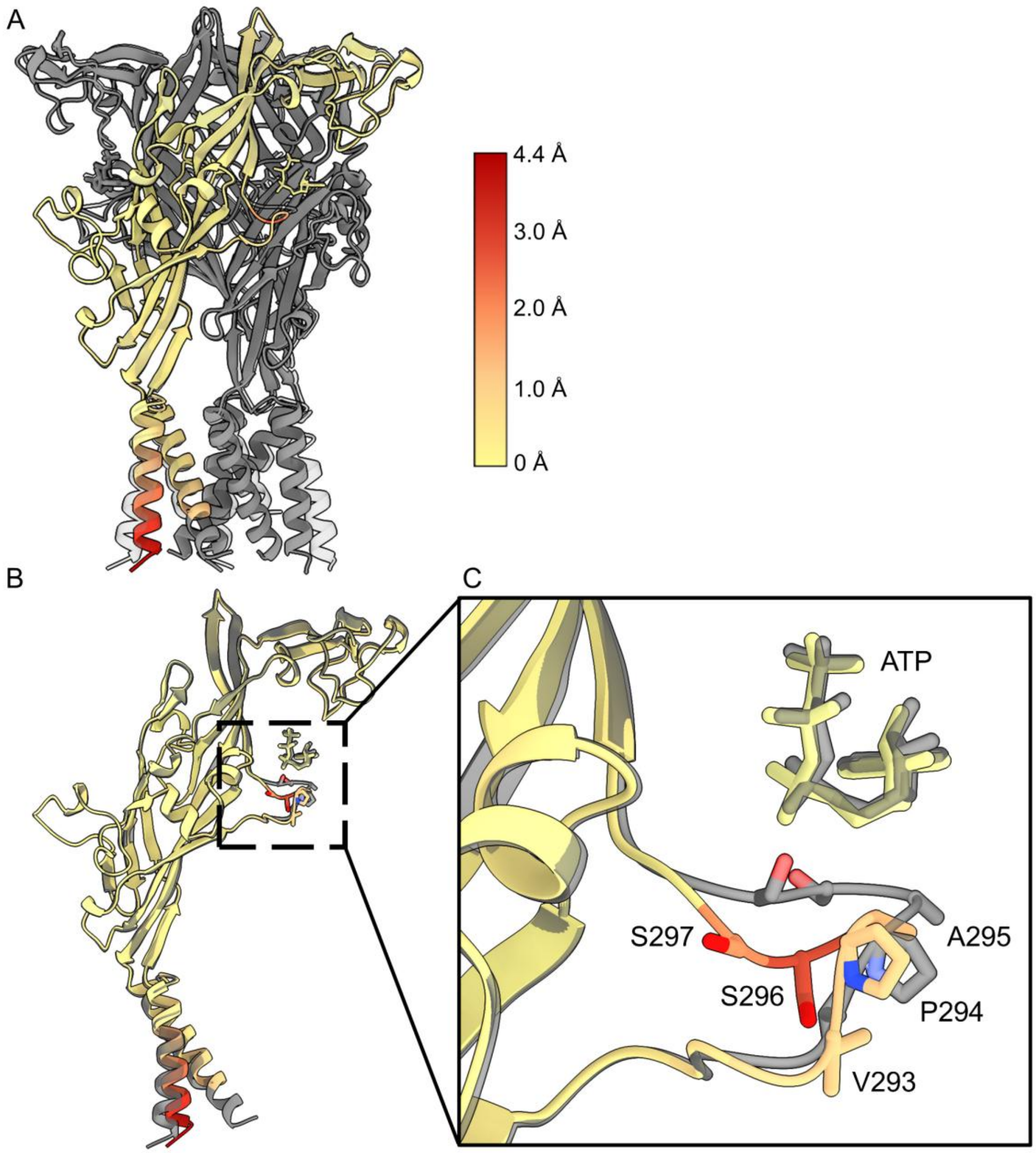

Fig. S12. Structural differences of the hP2X2R in two distinct ATP-bound desensitized states (conformation I and II). (*A*) Ribbon representation of the two ATP-bound desensitized state structures with conformation I in transparent gray and conformation II in gray. One protomer of conformation II is colored by RMSD according to the structural differences between conformation I and II. Residues colored in yellow signify little deviation with an RMSD of 0 Å while residues colored in red signify a larger deviation with an RMSD of > 4.4 Å. The RMSDs were calculated with ChimeraX (10, 11). Overall, the two states are very similar, but there are differences in the left flipper (RMSD > 2.0 Å) and TM1 (RMSD > 4.4 Å). (*B*) Ribbon representation of one protomer from each ATP-bound desensitized state conformation with conformation I in gray and conformation II colored by RMSD in the same scale as panel *A*. (*C*) Magnified view of the dotted box in panel *B* highlighting the differences in the left flipper between the two experimentally determined ATP-bound desensitized state structures (conformation I and II). Side chains for residues on the left flipper are colored by atom: nitrogen in blue and oxygen in red.

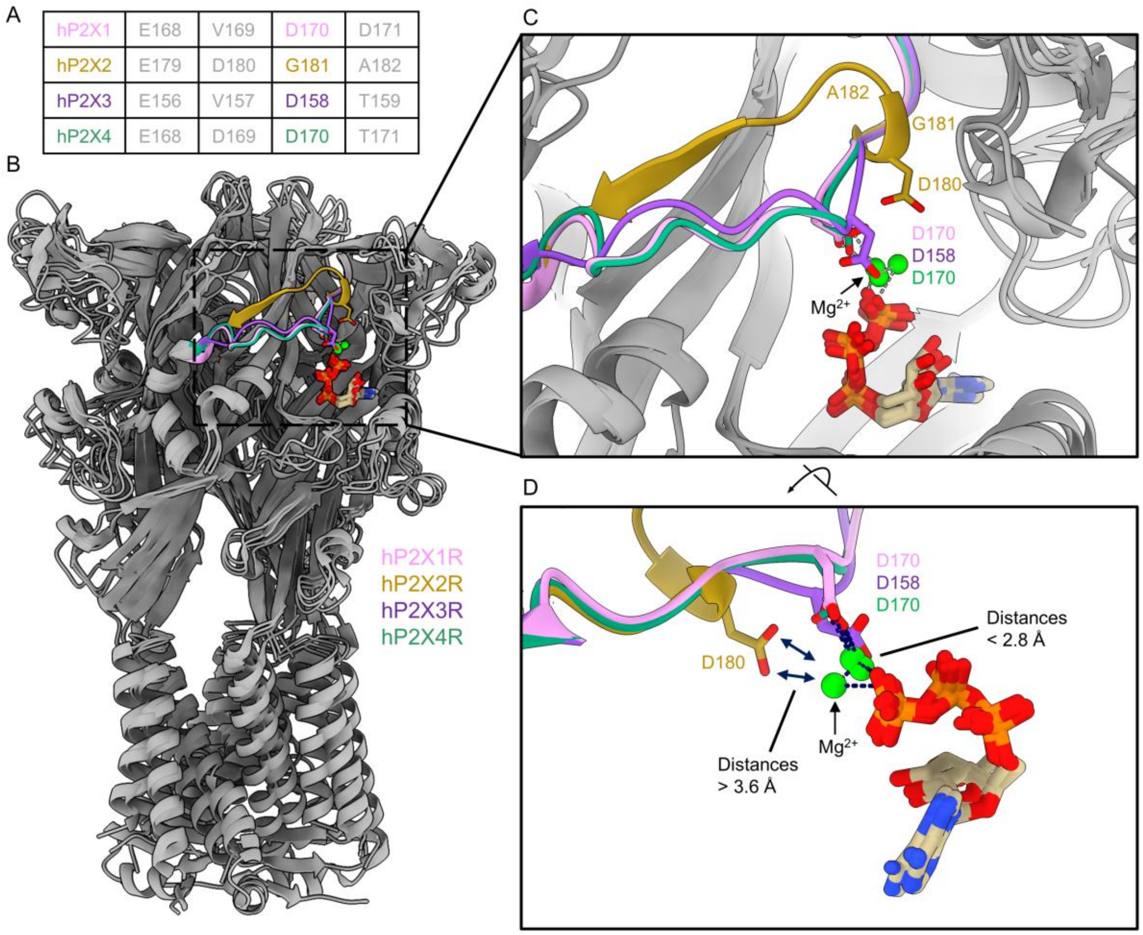

Fig. S13. Structural differences of the left flipper domain between different P2XR subtypes in the ATP-bound desensitized state. (*A*) Sequence alignment of a short stretch of residues on the left flipper between hP2X1R, hP2X2R, hP2X3R, and hP2X4R. (*B*) Aligned ribbon representation of hP2X1R, hP2X2R, hP2X3R, and hP2X4R structures in the canonical ATP-bound desensitized state highlighting differences in the region known to coordinate divalent cations. Within the black dotted box, receptors are colored by subtype: hP2X1R (pink, PDB ID: 9C2B (6)), hP2X2R conformation I (gold), hP2X3R (purple, PDB ID: 6AH5 (17)), and hP2X4R (green, PDB ID: 9C48 (7)). (*C*) Magnified view of panel *B* highlighting the structural differences in the region known to coordinate divalent cations. The hP2X2R is structurally very different from hP2X1R, hP2X3R, and hP2X4R all of which have Mg^{2+}-ions in their reported structures. (*D*) Further magnified and rotated (180° and 90° around x-axis and z-axis, respectively) view from panel *C* highlighting the coordination distances between the Mg^{2+}-ion and the aspartate residue that coordinates it across P2XR subtypes. Although an aspartate (D180) is present in hP2X2R in close proximity, the distance (> 3.6 Å) is too far to allow for coordination with $MgATP^{2-}$. The side chains shown and the heteroatoms of ATP are colored by atom: nitrogen in blue, oxygen in red, and phosphorus in orange. The carbon atoms of ATP are shown in tan.

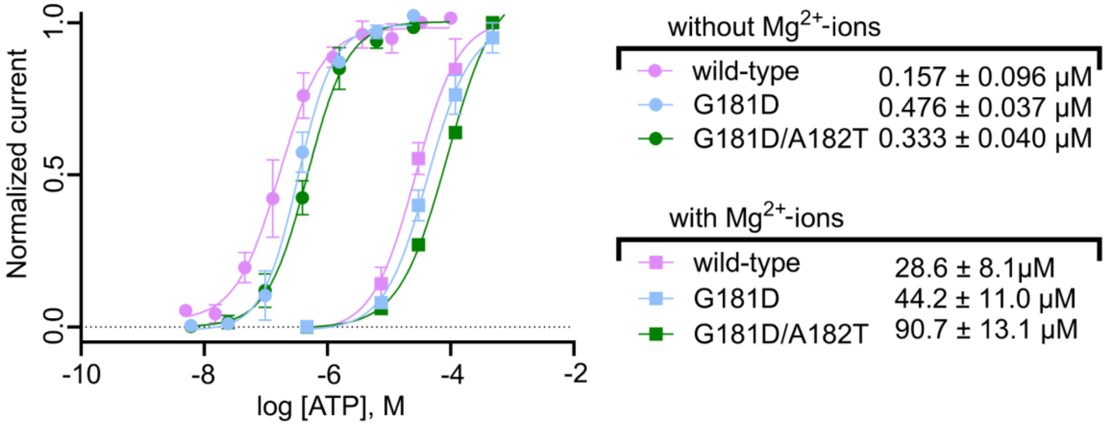

Fig. S14. Concentration-response curves for wild-type and mutant hP2X2Rs highlighting the impact of mutations, known to coordinate Mg^{2+}-ions in other P2XR subtypes, on the activation of the receptor. Concentration-response curves determined in TEVC experiments measuring ATP-induced activation of the wild-type hP2X2R, G181D mutant and G181D/A182T mutant. While divalent cation-free and Mg^{2+}-containing (5 mM) buffers affect the apparent affinity of the hP2X2R for ATP, it appears that the mutations in hP2X2R, that mimic the analogous residues in the hP2X3R and hP2X4R known to coordinate Mg^{2+}-ions, do not dramatically impact on the apparent affinity of ATP or the receptor´s Mg^{2+}-ion sensitivity. Data points and error bars represent mean ± standard deviation of normalized current, respectively, across triplicate experiments.

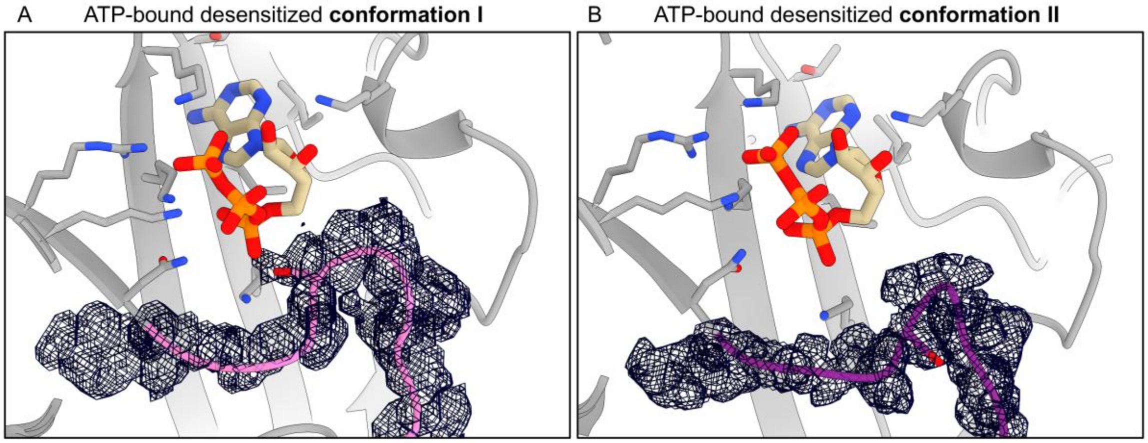

Fig. S15. Cryo-EM density supports two conformations of the left flipper. (*A* and *B*) Same view as in Fig. 3 showing the cryo-EM density for the left flipper in the ATP-bound desensitized state structure of (*A*) Conformation I (pink) and (*B*) Conformation II (purple). These panels highlight a distinct difference in the density surrounding the left flipper between the two conformations, including residue S296. The cryo-EM density is shown in black mesh. The side chains shown and the heteroatoms of ATP are colored by atom: nitrogen in blue, oxygen in red, and phosphorus in orange. The carbon atoms of ATP are shown in tan.

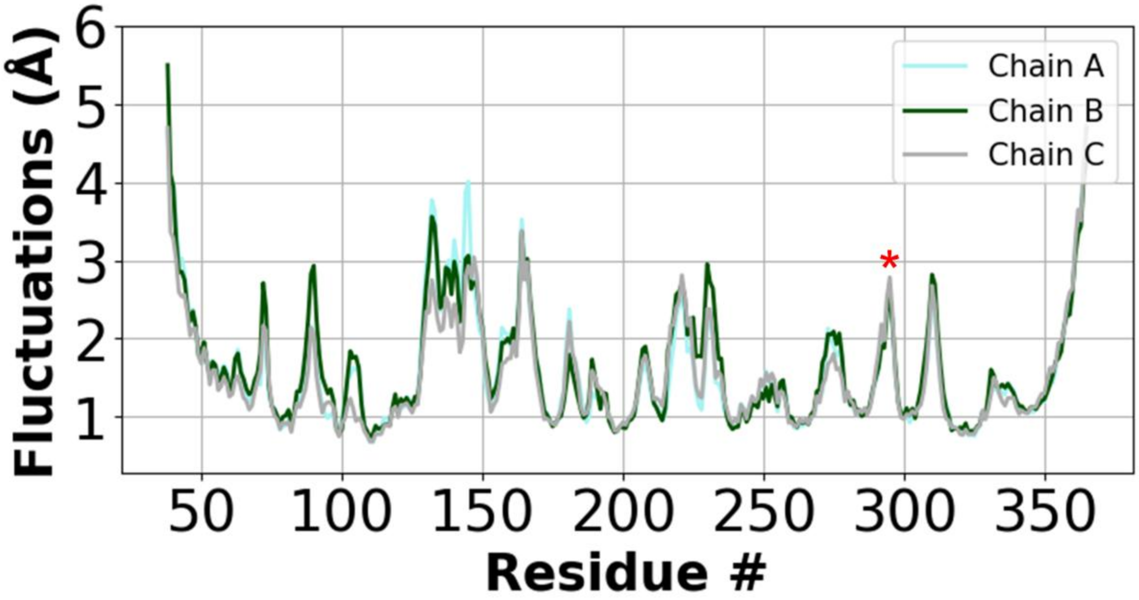

Fig. S16. Root-mean-square fluctuation (RMSF in Å, estimated for the Cα atoms) analysis of the trimeric hP2X2R in the apo state, based on 1-μs MD simulations. RMSF values are shown for chains A (light blue), B (green), and C (gray), averaged over five replicates. The region corresponding to the left flipper loop (residues 288–299) is highlighted with a red asterisk.

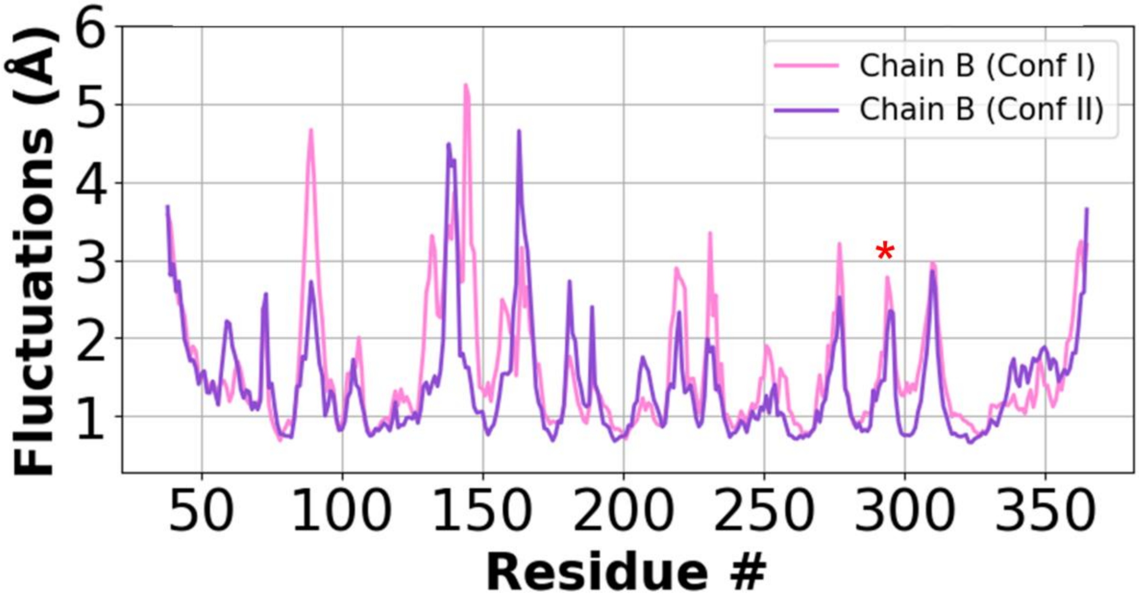

Fig. S17. Root-mean-square fluctuation (RMSF in Å, estimated for the Cα atoms) analysis of the trimeric hP2X2R in the two ATP-bound desensitized states (conformation I and conformation II), based on 1-μs MD simulations. RMSF values are shown for chain B (conformation I, pink) and (conformation II, purple), from a representative replicate. The peaks represent flexible regions in the protein. The region corresponding to the left flipper loop (residues 288–299) is highlighted with a red asterisk.

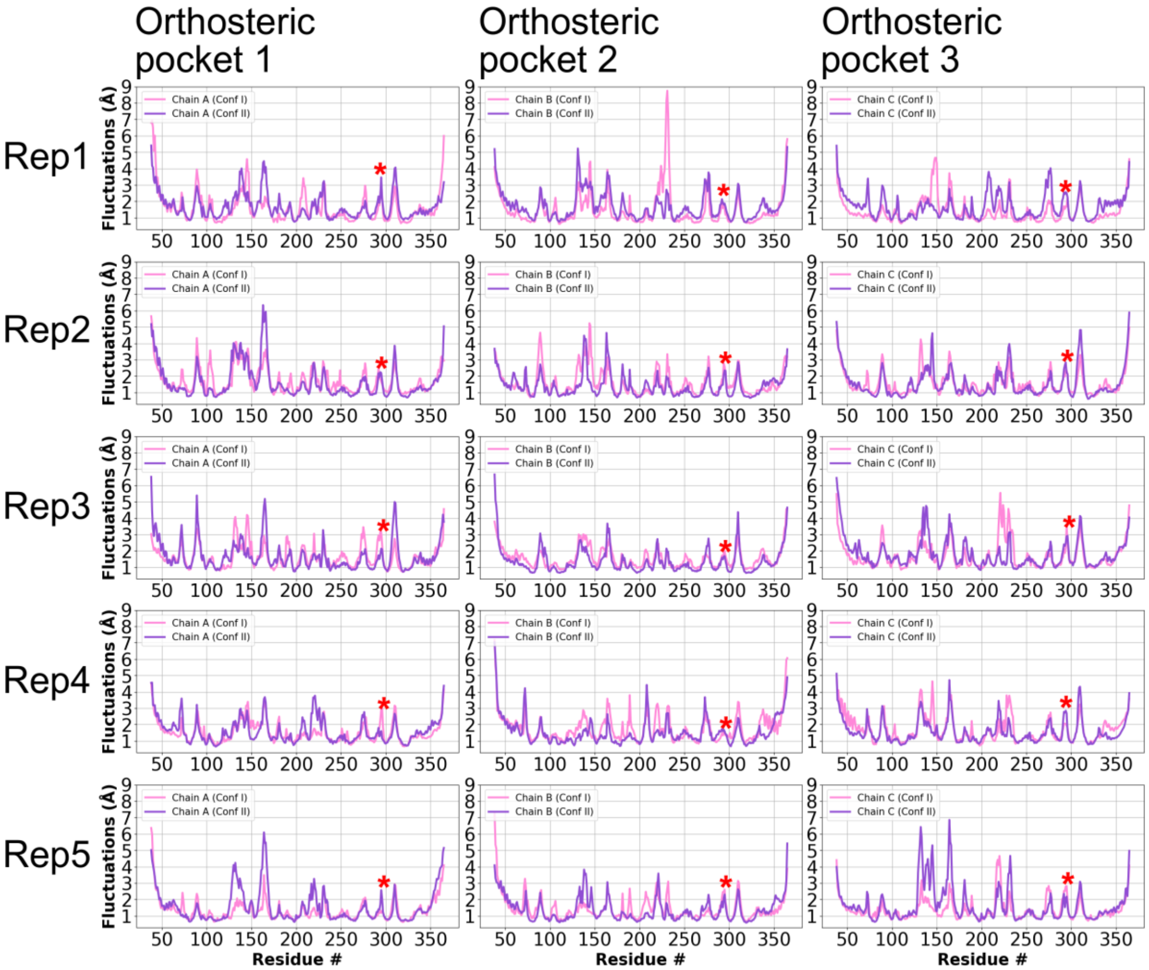

Fig. S18. Root-mean-square fluctuation (RMSF in Å, estimated for the Cα atoms) analysis of the trimeric hP2X2R in the two ATP-bound desensitized states (conformation I and conformation II) plotted for chains A-C based on 1-μs MD simulations for all replicates. MD simulations starting from conformation I are shown in pink and MD simulations starting from conformation II are shown in purple. The peaks represent flexible regions in the protein. The region corresponding to the left flipper loop (residues 288–299) is highlighted with a red asterisk. The analysis reveals an average fluctuation of S296 in the left flipper of > 2 Å (chain A: 2.38 ± 0.90 Å - conformation I and 2.39 ± 0.69 Å - conformation II, chain B: 2.04 ± 0.53 - conformation I and 1.96 ± 0.35 Å - conformation II, chain C: 2.45 ± 0.71 Å - conformation I and 2.49 ± 0.30 Å - conformation II). The fluctuation averages represent mean ± standard deviation across five replicates per chain. This suggests that in all five replicates the left-flipper loop exhibits similar flexibility during the simulations.

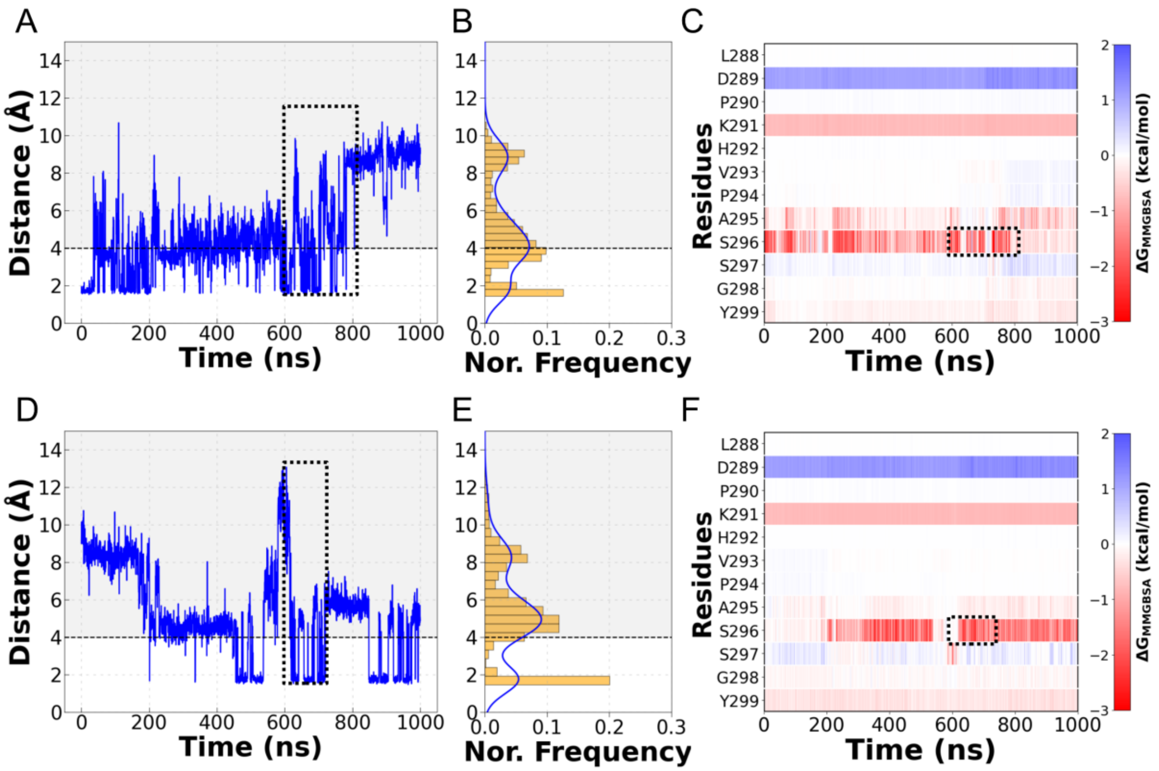

Fig. S19. Visualization of the 1-μs simulation studies performed for ATP-bound hP2X2R in the desensitized conformation I (A-C) and conformation II (D-F). (*A* and *D*) Visualizations for the distance (in Å) between the side chain hydroxyl group of S296 and the α-phosphate of ATP for one representative trajectory (*A*) starting from conformation I, and (*D*) starting from conformation II. (*B* and *E*) Distance measurements represented in Kernel Density Estimate (KDE, blue curve) and normalized frequency (orange bars). (*C* and *F*) Per-residue binding free energy ($\Delta G_{MM/GBSA}$) (kcal/mol) computed for the residues present in the left-flipper loop region using the Molecular Mechanics with Generalized Born Surface Area (MM/GBSA) method for one representative trajectory starting from conformation I (*C*) and one representative trajectory starting from conformation II. (*F*) The dotted black box in *A, C, D* and *F* represents the region where the respective movie (SI *Appendix* Movie S2 and S3) was rendered. The dotted line in *A, B, D*, and *E* at 4 Å indicates the threshold set to define a formed interaction of S296 with ATP in the binding pocket.

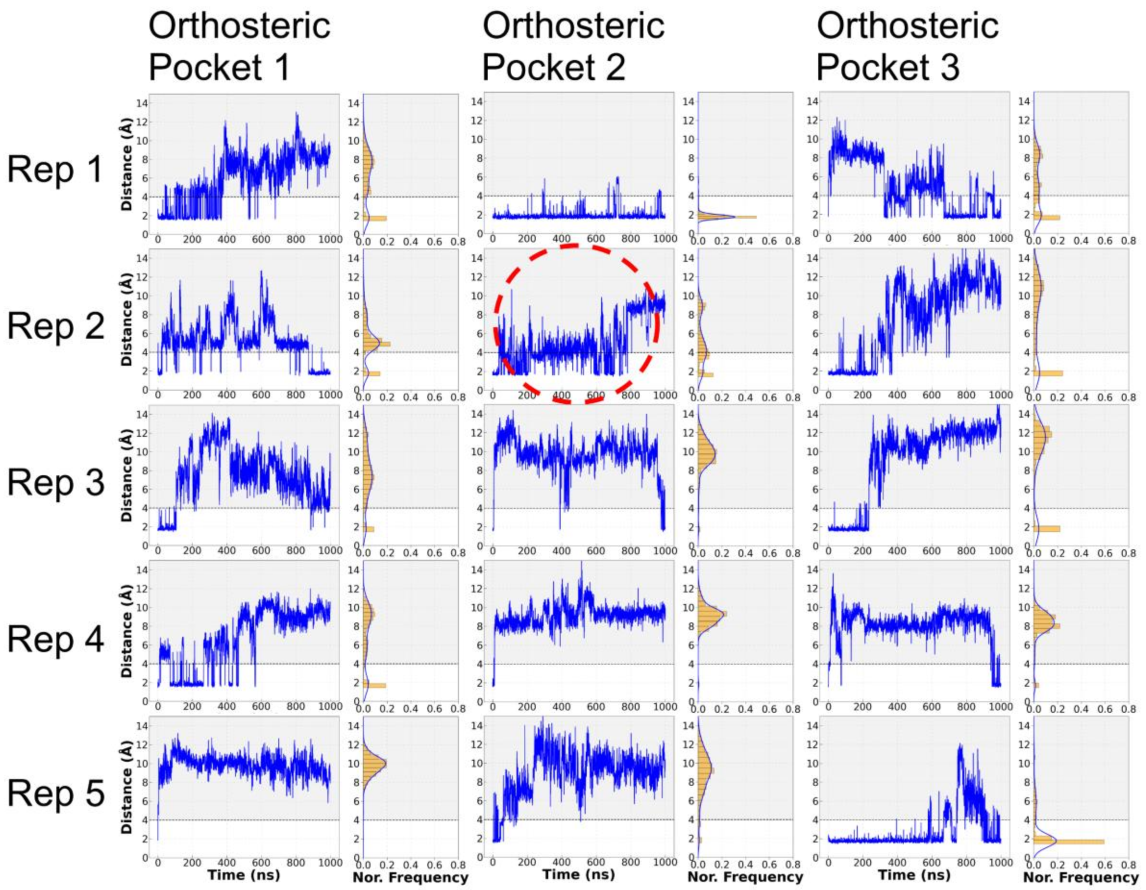

Fig. S20. Visualization of the trajectories of the MD simulations starting from the canonical ATP-bound desensitized state conformation I. The distances (in Å) between the side chain hydroxyl group of S296 and the α-phosphate of ATP are visualized. Trajectories of all five replicates (Rep1-Rep5) are shown. Orthosteric pockets 1–3 represent the three ATP binding sites of the hP2X2R. The corresponding histograms show distance measurements, with Kernel Density Estimates (KDE, blue curve) and normalized frequency (orange bars). The dotted circle (red) highlights the representative trajectory depicted in SI Fig. S19*A*.

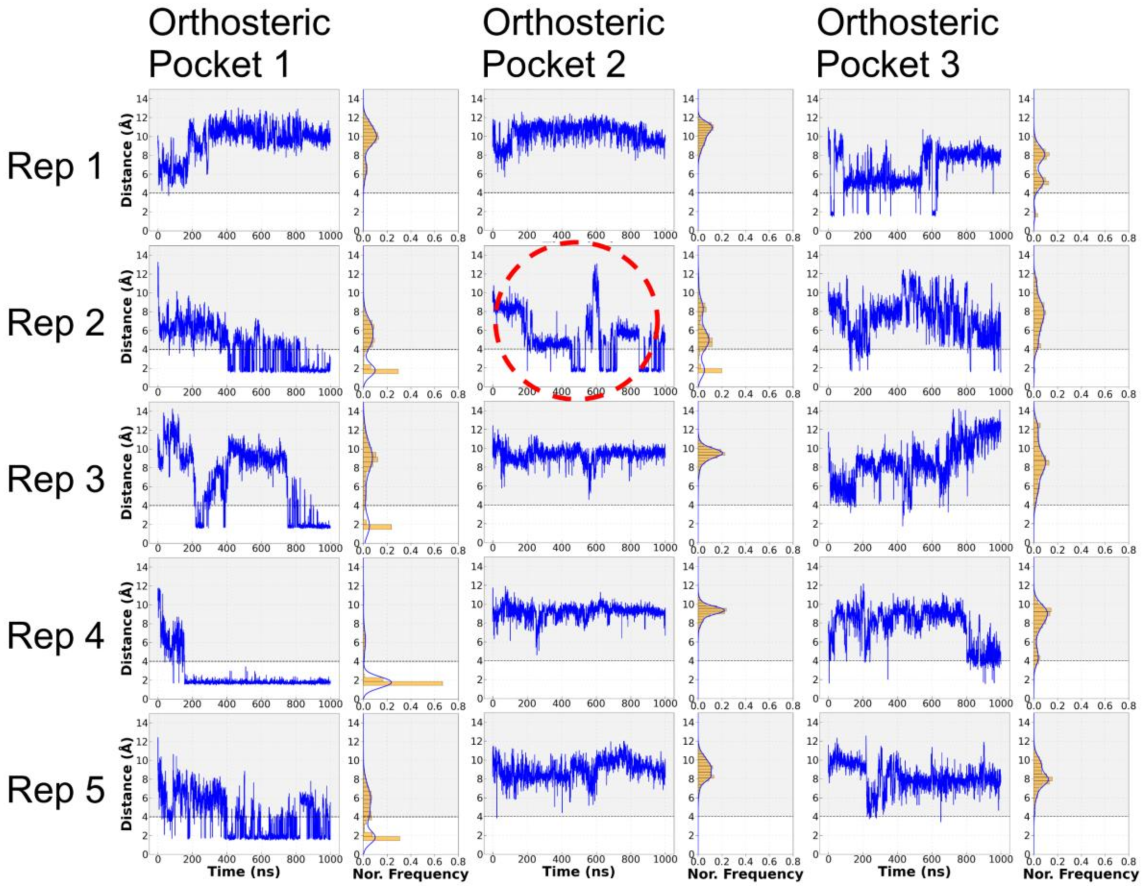

Fig. S21. Visualization of the trajectories of the MD simulations starting from the alternate ATP-bound desensitized state conformation II. The distances (in Å) between the side chain hydroxyl group of S296 and the α-phosphate of ATP are visualized. Trajectories of all five replicates (Rep1-Rep5) are shown. Orthosteric pockets 1–3 represent the three ATP binding sites of the hP2X2R. The corresponding histograms show distance measurements, with Kernel Density Estimates (KDE, blue curve) and normalized frequency (orange bars). The dotted circle (red) highlights the representative trajectory depicted in SI Fig. S19*D* and S25*B*.

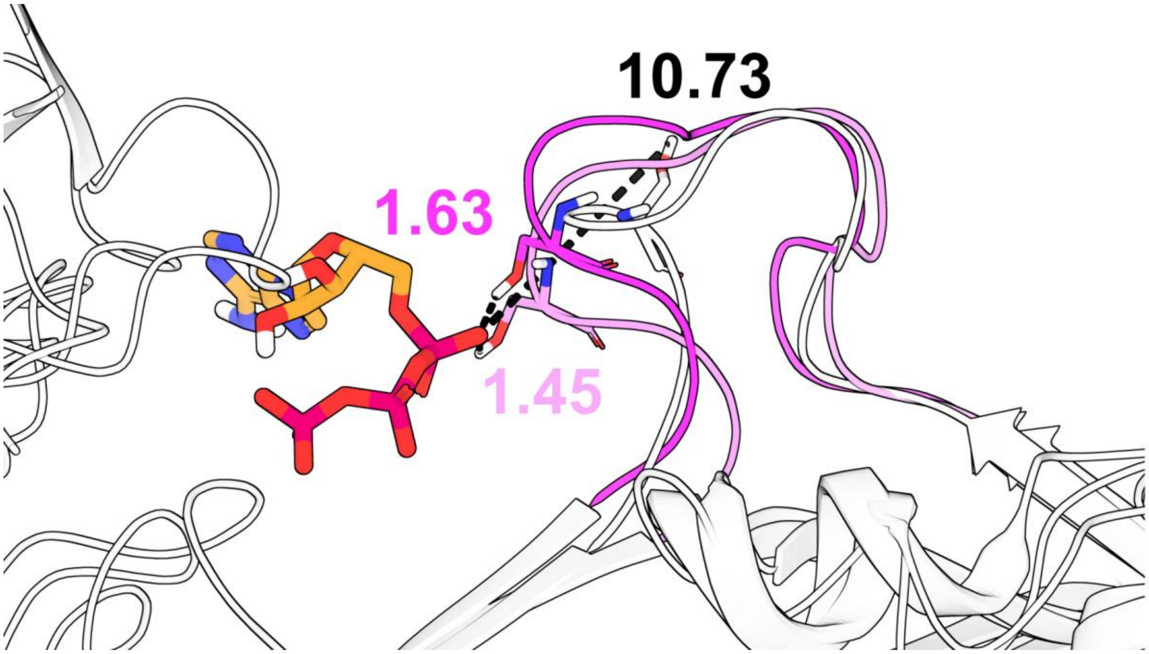

Fig. S22. The left flipper is dynamic in the MD simulations. Close-up view showing three different conformations of the left-flipper loop (residues 288–299, represented as tubes) of chain B of the hP2X2R (white ribbon) observed during one representative MD simulation trajectory (*SI Appendix,* Fig. S19*A*) starting from the ATP-bound desensitized state conformation I. The distance between the side chain hydroxyl group of S296 and the α-phosphate of ATP (dotted black lines) was measured and reported to highlight the dynamic nature of the loop. The snapshots show the initial frame (pink, 1.63 Å - 0 ns), the minimum distance (light pink, 1.45 Å - 698 ns) and the maximum distance (white, 10.73 Å – 887 ns) that could be measured in this trajectory. More conformations of the left flipper were observed (see *SI Appendix*, Movie S2). The side chains shown and the heteroatoms of ATP are depicted in stick representation and colored by atom: nitrogen in blue, oxygen in red, hydrogen in white, and phosphorus in magenta. The carbon atoms of ATP are shown in tan.

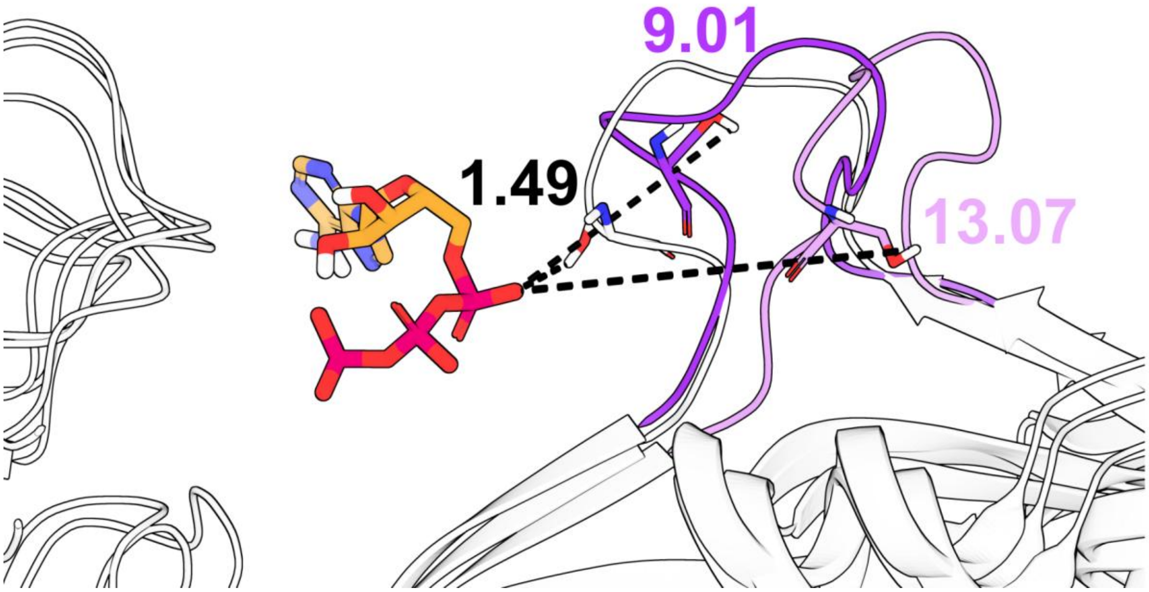

Fig. S23. The left flipper is dynamic in the MD simulations. Close-up view showing three different conformations of the left-flipper loop (residues 288–299, represented as tubes) of chain B of the hP2X2R (white ribbon) observed during one representative MD simulation trajectory (*SI Appendix,* Fig. S19*D*) starting from the ATP-bound desensitized state conformation II. The distance between the side chain hydroxyl group of S296 and the α-phosphate of ATP (dotted black lines) was measured and reported to highlight the dynamic nature of the loop. The snapshots show the initial frame (purple, 9.01 Å - 0 ns), the minimum distance (white, 1.49 Å - 964 ns) and the maximum distance (light purple, 13.07 Å – 605 ns) that could be measured in this trajectory. More conformations of the left flipper were observed (see *SI Appendix*, Movie S3). The side chains shown and the heteroatoms of ATP are depicted in stick representation and colored by atom: nitrogen in blue, oxygen in red, hydrogen in white, and phosphorus in magenta. The carbon atoms of ATP are shown in tan.

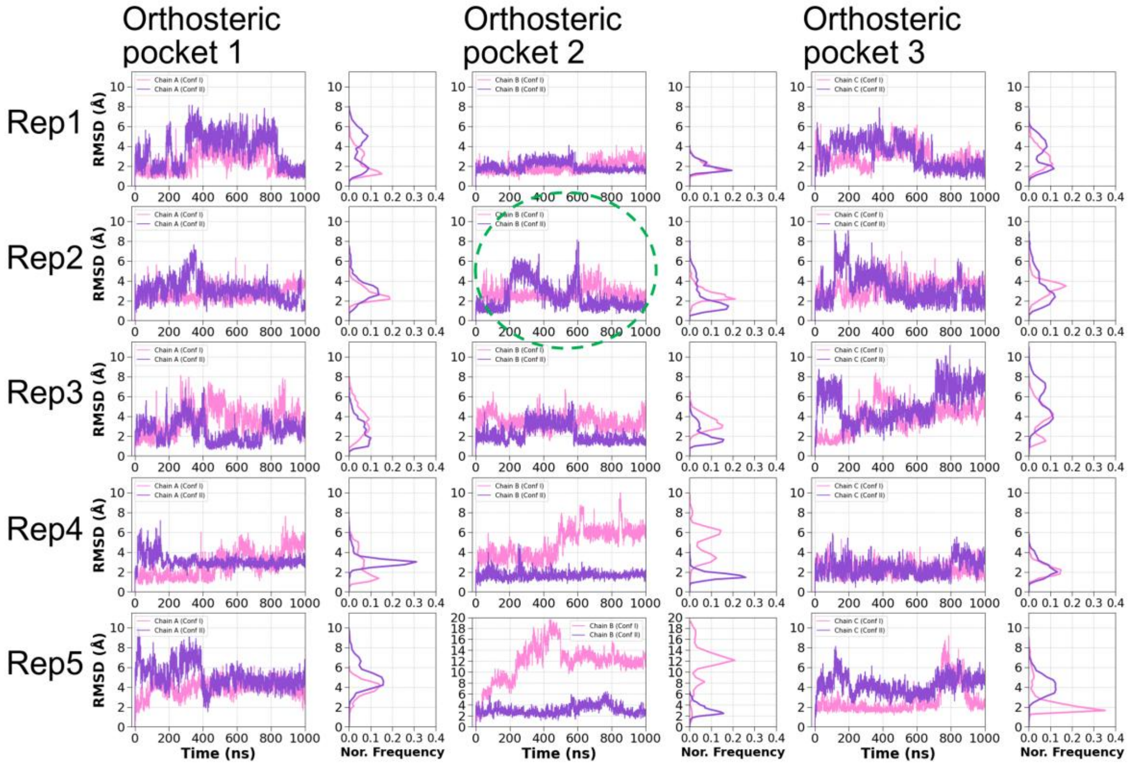

Fig. S24. Root-mean-square deviation (RMSD) analysis for ATP in the orthosteric pocket of the hP2X2R over time. The analysis is based on 1-μs MD simulations initiated from ATP-bound desensitized conformation I (pink), and conformation II (purple). The timeline of ATP RMSDs and the normalized frequency distributions of the ATP RMSD values (in Å) for all five replicates (Rep1-Rep5) are shown. Orthosteric pockets 1–3 represent the three ATP binding sites of the hP2X2R. The corresponding histograms show distance measurements, with Kernel Density Estimates (KDE, blue curve) and normalized frequency (orange bars). The trajectory in the dotted circle (green) is shown as representative in SI *Appendix*, Fig. S25*B*.

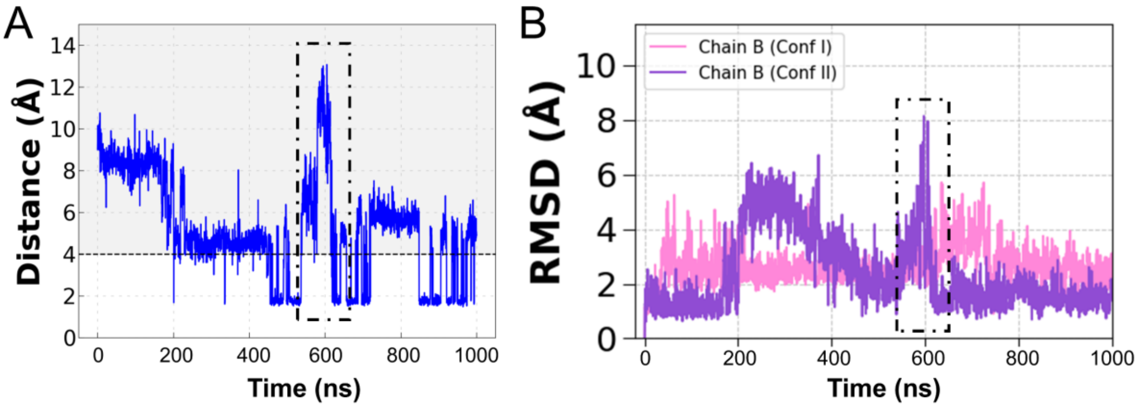

Fig. S25. Distance measurements of S296 and ATP compared to the root-mean-square deviation (RMSD) analysis for ATP in the orthosteric pocket of the hP2X2R of the same representative trajectory based on a 1-µs MD simulation. (*A*) Visualization of the distances (in Å) between the side chain hydroxyl group of S296 and the α-phosphate of ATP starting from conformation II. (*B*) The timeline of ATP RMSD (in Å) for conformation I (pink) and conformation II (purple). For this trajectory it can be observed that a rapid loss of the S296-ATP interaction causes an increased RMSD of the ATP in the orthosteric pocket (dashed black box). This trend can be observed for many but not all replicates (see *SI Appendix*, Fig. S24). However, this indicates that ATP in the binding pocket might maintain a more stable pose when the S296-ATP interaction is formed.

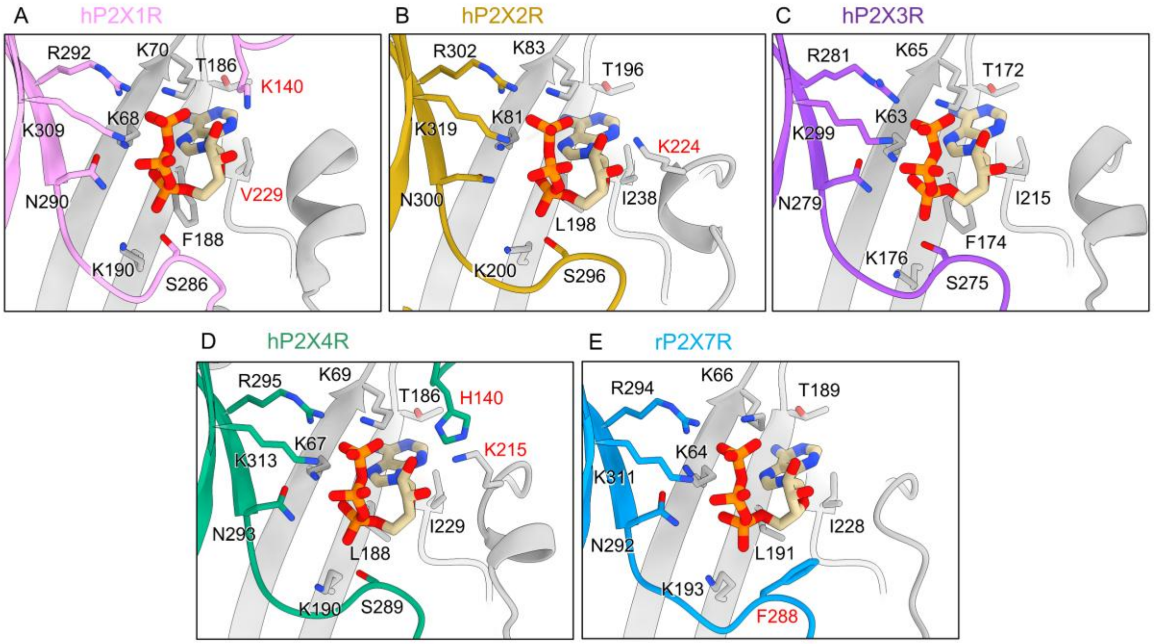

Fig. S26. A comparison of the ATP-bound orthosteric pockets between P2XR subtypes. (*A*-*E*) Comparison of ATP bound in the orthosteric pocket of each P2XR subtype for which a structure has been published. Conserved residues that interact with ATP are labeled in black and non-conserved residues are highlighted with red labels. (*A*) hP2X1R (pink and light gray, PDB ID: 9C2B (6)) (*B*) hP2X2R - conformation I (gold and light gray), (*C*) hP2X3R (purple and light gray, PDB ID: 5SVK (8)), (*D*) hP2X4R (green and light gray, PDB ID: 9C48 (7)), and (*E*) rP2X7R (blue and light gray, PDB ID: 6U9W (18)). Residues labeled in red are not conserved among all P2XR subtypes shown. The side chains shown and the heteroatoms of ATP are colored by atom: nitrogen in blue, oxygen in red, and phosphorus in orange. The carbon atoms of ATP are shown in tan.

Table S1. Cryo-EM collection, refinement, and validation statistics for hP2X2R datasets.

	Closed apo state (EMD-46781) (PDB: 9DDV)	ATP-bound desensitized Conformation I (EMD-46782) (PDB: 9DDW)	ATP-bound desensitized Conformation II (EMD-46783) (PDB: 9DDX)
**Data collection and processing**			
Magnification (kx)	130	130	130
Voltage (kV)	300	300	300
Electron exposure (e–/Å^2)	45	45	45
Movie frames	50	50	50
Defocus range (µm)	-0.9 to -1.5	-0.9 to -1.5	-0.9 to -1.5
Pixel size (Å)	0.648 (0.324 super-res)	0.648 (0.324 super-res)	0.648 (0.324 super-res)
Symmetry imposed	C3	C3	C3
Initial micrographs (no.)	27,324 + 6,000	15,645	27,324
Final micrographs used (no.)	26,607 + 6,000	13,068	26,607
Initial particle images (no.)	3,372,653 + 879,653	1,842,703	3,372,653
Final particle images (no.)	231,214	411,445	108,423
Map resolution (Å)<br>FSC threshold	2.71<br>(0.143)	2.49<br>(0.143)	2.55<br>(0.143)
Map resolution range (Å)	1.6-33.5	2.2-40.5	1.5 - 31.2
**Refinement**			
Initial model used (PDB code)	Homology model: 4DW0	Homology model: 6U9W	Homology model: 6U9W
Model resolution (Å)<br>FSC threshold	2.71<br>(0.143)	2.48<br>(0.143)	2.53<br>(0.143)
Map sharpening B factor (Å^2)	95.2	100	89.4
**Model composition**			
Non-hydrogen atoms	7,866	7647	7767
Protein Residues	981	939	957
Ligands	6	9	9
Waters	180	159	147
**B factors (Å^2)**			
Protein	43.5/93.5/55.9	46.2/138/59.0	16.0/111/48.3
Ligand	59.6/84.9/72.1	49.8/84.659.9	33.0/93.0/59.1
Nucleotide	--	--	--
Water	45.3/64.0/53.8	47.0/62.0/53.0	41.0/53.7/46.4
**R.m.s. deviations**			
Bond lengths (Å)	0.005 (0)	0.007 (0)	0.007 (0)
Bond angles (°)	0.692 (0)	0.956 (0)	1.252 (0)
**Validation**			
MolProbity score	1.56	1.26	1.18
Clash score	4.60	5.00	3.93
Poor rotamers (%)	0.00	0.00	0.00
**Ramachandran plot**			

Favored (%)	95.38	98.07	98.11
Allowed (%)	4.62	1.93	1.89
Disallowed (%)	0.00	0.00	0.00

Movie S1 (separate file). Visualization of one representative MD simulation for the hP2X2R in the apo closed state highlighting the dramatic conformational flexibility of the left flipper in the absence of ATP. The movie shows the movements between 540 ns and 800 ns of the representative trajectory. The backbone of the left flipper (residues 288-299) is depicted in orange, with the residues S296, P294, and P290 shown in stick representation. The heteroatoms are shown in color: nitrogen in blue and oxygen in red. Hydrogen atoms are shown in white. Neighboring protein domains are represented by contours in black.

Movie S2 (separate file). Visualization of one representative MD simulation for the hP2X2R in the canonical ATP-bound desensitized state (conformation I) highlighting the movements of residue S296 and ATP. The movie shows the movements between 600 ns and 800 ns of one representative trajectory. The backbone of the left flipper (residues 288-299) is depicted in blue, with the residues S296, P294, and P290 shown in stick representation. The heteroatoms are shown in color: nitrogen in blue, oxygen in red, and phosphorus in magenta. Hydrogen atoms are shown in white and carbon atoms of ATP in tan. Neighboring protein domains are represented by contours in black. Distances between S296 and the α-phosphate of ATP are labeled in green.

Movie S3 (separate file). Visualization of one representative MD simulation for the hP2X2R in the alternate ATP-bound desensitized state (conformation II) highlighting the movements of residue S296 and ATP. The movie shows the movements between 600 ns and 750 ns of one representative trajectory. The backbone of the left flipper (residues 288-299) is depicted in dark green, with the residues S296, P294, and P290 shown in stick representation. The heteroatoms are shown in color: nitrogen in blue, oxygen in red, and phosphorus in magenta. Hydrogen atoms are shown in white and carbon atoms of ATP in tan. Neighboring protein domains are represented by contours in black. Distances between S296 and the α-phosphate of ATP are labeled in green.

References

1. A. Fiser, A. Sali, Modeller: generation and refinement of homology-based protein structure models. *Methods Enzymol.* **374**, 461–491 (2003).

2. A. M. Waterhouse, J. B. Procter, D. M. A. Martin, M. Clamp, G. J. Barton, Jalview Version 2--a multiple sequence alignment editor and analysis workbench. *Bioinformatics* **25**, 1189–1191 (2009).

3. J. Li, R. Abel, K. Zhu, Y. Cao, S. Zhao, R. A. Friesner, The VSGB 2.0 model: a next generation energy model for high resolution protein structure modeling. *Proteins* **79**, 2794–2812 (2011).

4. A. Punjani, J. L. Rubinstein, D. J. Fleet, M. A. Brubaker, cryoSPARC: algorithms for rapid unsupervised cryo-EM structure determination. *Nat. Methods.* **14**, 290–296 (2017).

5. T. Kawate, J. C. Michel, W. T. Birdsong, E. Gouaux, Crystal structure of the ATP-gated P2X(4) ion channel in the closed state. *Nature* **460**, 592–598 (2009).

6. A. C. Oken, N. E. Lisi, I. A. Ditter, H. Shi, N. A. Nechiporuk, S. E. Mansoor, Cryo-EM structures of the human P2X1 receptor reveal subtype-specific architecture and antagonism by supramolecular ligand-binding. *Nat. Commun.* **15 (**2024).

7. H. Shi, I. A. Ditter, A. C. Oken, S. E. Mansoor, Human P2X4 receptor gating is modulated by a stable cytoplasmic cap and a unique allosteric pocket. *Sci. Adv.* **11**, eadr3315 (2025).

8. S. E. Mansoor, W. Lü, W. Oosterheert, M. Shekhar, E. Tajkhorshid, E. Gouaux, X-ray structures define human P2X(3) receptor gating cycle and antagonist action. *Nature* **538**, 66–71 (2016).

9. M. Hattori, E. Gouaux, Molecular mechanism of ATP binding and ion channel activation in P2X receptors. *Nature* **485**, 207–212 (2012).

10. E. C. Meng, T. D. Goddard, E. F. Pettersen, G. S. Couch, Z. J. Pearson, J. H. Morris, T. E. Ferrin, UCSF ChimeraX: Tools for structure building and analysis. *Protein Sci.* **32**, e4792 (2023).

11. E. F. Pettersen, T. D. Goddard, C. C. Huang, E. C. Meng, G. S. Couch, T. I. Croll, J. H. Morris, T. E. Ferrin, UCSF ChimeraX: Structure visualization for researchers, educators, and developers. *Protein Sci.* **30**, 70–82 (2021).

12. J. Abramson, et al., Accurate structure prediction of biomolecular interactions with AlphaFold 3. *Nature* **630**, 493–500 (2024).

13. V. Le Guilloux, P. Schmidtke, P. Tuffery, Fpocket: an open source platform for ligand pocket detection. *BMC bioinformatics* **10**, 168 (2009).

14. A. C. Oken, N. E. Lisi, I. Krishnamurthy, A. E. McCarthy, M. H. Godsey, A. Glasfeld, S. E. Mansoor, High-affinity agonism at the P2X7 receptor is mediated by three residues outside the orthosteric pocket. *Nat. Commun.* **15**, 6662 (2024).

15. P. Illes, C. E. Müller, K. A. Jacobson, T. Grutter, A. Nicke, S. J. Fountain, C. Kennedy, G. Schmalzing, M. F. Jarvis, S. S. Stojilkovic, B. F. King, F. Di Virgilio, Update of P2X receptor properties and their pharmacology: IUPHAR Review 30. *Br. J. Pharmacol.* **178**, 489–514 (2021).

16. B. George, K. J. Swartz, M. Li, Hearing loss mutations alter the functional properties of human P2X2 receptor channels through distinct mechanisms. *Proc. Natl. Acad. Sci. U.S.A.* **116**, 22862–22871 (2019).

17. M. Li, Y. Wang, R. Banerjee, F. Marinelli, S. Silberberg, J. D. Faraldo-Gómez, M. Hattori, K. J. Swartz, Molecular mechanisms of human P2X3 receptor channel activation and modulation by divalent cation bound ATP. *eLife* **8 (**2019).

18. A. E. McCarthy, C. Yoshioka, S. E. Mansoor, Full-Length P2X7 Structures Reveal How Palmitoylation Prevents Channel Desensitization. *Cell* **179**, 659-670.e13 (2019).
